# *Bacillus thuringiensis* and *Bacillus weihenstephanensis* Inhibit the Growth of Phytopathogenic *Verticillium* Species

**DOI:** 10.3389/fmicb.2016.02171

**Published:** 2017-01-18

**Authors:** Jacqueline Hollensteiner, Franziska Wemheuer, Rebekka Harting, Anna M. Kolarzyk, Stefani M. Diaz Valerio, Anja Poehlein, Elzbieta B. Brzuszkiewicz, Kai Nesemann, Susanna A. Braus-Stromeyer, Gerhard H. Braus, Rolf Daniel, Heiko Liesegang

**Affiliations:** ^1^Göttingen Genomics Laboratory, Department of Genomic and Applied Microbiology, Institute of Microbiology and Genetics, Georg-August-UniversityGottingen, Germany; ^2^Department of Molecular Microbiology and Genetics, Institute of Microbiology and Genetics and Göttingen Center for Molecular Biosciences, Georg-August-UniversityGottingen, Germany

**Keywords:** *Bacillus thuringiensis*, *Bacillus weihenstephanensis*, *Verticillium*, bacterial-fungal interaction, antifungal activity, biopesticide, plant pathogen, chitinase

## Abstract

*Verticillium* wilt causes severe yield losses in a broad range of economically important crops worldwide. As many soil fumigants have a severe environmental impact, new biocontrol strategies are needed. Members of the genus *Bacillus* are known as plant growth-promoting bacteria (PGPB) as well as biocontrol agents of pests and diseases. In this study, we isolated 267 *Bacillus* strains from root-associated soil of field-grown tomato plants. We evaluated the antifungal potential of 20 phenotypically diverse strains according to their antagonistic activity against the two phytopathogenic fungi *Verticillium dahliae* and *Verticillium longisporum*. In addition, the 20 strains were sequenced and phylogenetically characterized by multi-locus sequence typing (MLST) resulting in 7 different *Bacillus thuringiensis* and 13 *Bacillus weihenstephanensis* strains. All *B. thuringiensis* isolates inhibited *in vitro* the tomato pathogen *V. dahliae* JR2, but had only low efficacy against the tomato-foreign pathogen *V. longisporum* 43. All *B. weihenstephanensis* isolates exhibited no fungicidal activity whereas three *B. weihenstephanensis* isolates showed antagonistic effects on both phytopathogens. These strains had a rhizoid colony morphology, which has not been described for *B. weihenstephanensis* strains previously. Genome analysis of all isolates revealed putative genes encoding fungicidal substances and resulted in identification of 304 secondary metabolite gene clusters including 101 non-ribosomal polypeptide synthetases and 203 ribosomal-synthesized and post-translationally modified peptides. All genomes encoded genes for the synthesis of the antifungal siderophore bacillibactin. In the genome of one *B. thuringiensis* strain, a gene cluster for zwittermicin A was detected. Isolates which either exhibited an inhibitory or an interfering effect on the growth of the phytopathogens carried one or two genes encoding putative mycolitic chitinases, which might contribute to antifungal activities. This indicates that chitinases contribute to antifungal activities. The present study identified *B. thuringiensis* isolates from tomato roots which exhibited *in vitro* antifungal activity against *Verticillium* species.

## Introduction

*Verticillium* wilt occurs in a wide range of plant species including economical important crops. Symptoms include wilting, stunting, vascular discoloration, and early senescence, which cause an annual yield loss of billion dollars worldwide (Pegg and Brady, 2002; Fradin and Thomma, 2006). Causative agents are different soil-borne *Verticillium* species such as *Verticillium dahliae* and *Verticillium longisporum*. *Verticillium longisporum* has a narrow host range mainly infecting *Brassicaceae* (Zeise and von Tiedemann, 2002). Wilting symptoms or crop losses are only observed in the greenhouse (Zeise and von Tiedemann, 2002). In contrast, *V. dahliae* is known as a vascular pathogen with a broad host range including trees, legumes, ornamental crops, and vegetables such as tomato (Pegg and Brady, 2002; Luo et al., 2014). This fungus forms extremely outlasting melanized resting structures (microsclerotia) that are able to survive in soil for many years (Schnathorst, 1981). As consequence, the control of this phytopathogenic fungus is difficult. As many soil fumigants have a severe environmental impact, no pesticide which meets the standards of a sustainable agriculture is currently available to control the expansion of *V. dahliae* and *V. longisporum* (Frank, 2003; Depotter et al., 2016). Other control strategies such as crop rotation, the usage of resistant plant species, and soil solarization have only limited efficiency (Haas and Défago, 2005; Depotter et al., 2016). They are either ineffective, time-consuming, costly, may have a severe environmental impact, or even could affect human health (Angelopoulou et al., 2014).

Many plant-associated bacteria have beneficial effects on their host plant by increasing nutrient availability (Scherling et al., 2009) or by modulating the plant immunity (Jones and Dangl, 2006). Moreover, some of these plant growth-promoting bacteria (PGPB) have been used as biological control agents against plant diseases and pests (Ahemad and Kibret, 2014). However, common fungal antagonistic bacteria such as *Pseudomonas* species have only limited inhibitory impact on *Verticillium* due to the long-term persistence of *Verticillium* microsclerotia in soil (Angelopoulou et al., 2014). Members of the endospore-forming genus *Bacillus* possess a high potential as new fungal antagonists. They provide several advantages compared to other biocontrol agents such as (i) a better life-shell, (ii) a dry-product formulation which contains a lower contamination rate, (iii) established large-scale treatment, and finally, (iv) a cheap and easy usage (Katan, 1981; Fravel, 2005; Haas and Défago, 2005). Some *Bacilli* synthesize antifungal compounds such as cyclic lipopeptides produced by non-ribosomal peptide synthetases (NRPS), polyketide synthases (PKS) or mycolytic enzymes like chitinases (Swiontek Brzezinska et al., 2014; Aleti et al., 2015). Three families of *Bacillus* lipopeptides are known to confer an antifungal effect: surfactins, iturins, and fengycins (Ongena and Jacques, 2008). Strains of the species *Bacillus thuringiensis* (Bt) have been used as biological control agent against insecticidal crop pests for decades (Schnepf et al., 1998).

*Bacillus thuringiensis* (Bt) is a member of the *Bacillus cereus sensu lato* (Bcsl) complex, which comprises seven species (Priest et al., 2004) including the well-investigated name-giving species *B. cereus* (Bc), an opportunistic human pathogen. Other Bcsl members are *B. anthracis* (Ba), the etiological agent of anthrax, and *B. weihenstephanensis* (Bw). The latter is the only described species, which is able to grow under psychrophilic conditions (Lechner et al., 1998; Helgason et al., 2000). All Bcsl members share a highly conserved chromosomal backbone and have diverse extra-chromosomal elements (Priest et al., 2004). Especially Ba, Bc, and Bt strains showed high identity in a previous study (Priest et al., 1994). Despite their obvious similarity, specific phenotypic characteristics including the presence or absence of virulence genes have been used for differentiation (Helgason et al., 2000). However, the grouping of those species is in a still ongoing debate and it was suggested to reclassify this group to a single species (Okinaka and Keim, 2016). Bt is able to produce parasporal protein crystals consisting of δ-endotoxins (Schnepf et al., 1998). The insecticidal effect of entomopathogenic Bt is attributed to the production of these crystal toxins. In addition to its insecticidal effects, Rocha et al. (2014) reported that Bt serovar *kurstaki* can prevent the growth of the maize pathogen *Fusarium verticilloides*. However, a systematic investigation on different Bt strains isolated from root-associated soil as antagonists of *Verticillium* species has not been performed to date.

The objective of this study was to isolate members of the genus *Bacillus* to investigate their antifungal potential against two important phytopathogenic fungi differing in their host ranges. For this purpose, we generated a *Bacillus* strain collection of 267 isolates from tomato root-associated soil by using an enrichment method for Bt. Twenty *Bacillus* strains from this collection were selected based on unique morphological traits and further investigated for their antifungal activity against *V. dahliae* and *V. longisporum*. The phenotypic and taxonomic classification of the bacterial isolates was determined within the Bcsl species complex. The genomes of the 20 selected strains were sequenced and mined for genes related to crystal toxin as well as antifungal compound production. Moreover, the genome sequences were screened for cyclic lipopeptides produced by NRPS/PKS clusters and mycolytic enzymes such as chitinases. The abundance of the genera within the root-associated bacterial community composition of tomato plants was determined based on the bacterial 16S rRNA amplicon sequencing.

## Materials and methods

### Soil sampling and extraction of environmental DNA

Soil samples of *Solanum lycopersicum* were collected in a field plot near Göttingen (Germany, 51° 32′N, 9° 56′O) in June 2014. Samples of topsoil with 10 g each were taken next to roots (<3–5 mm) of three tomato plants and subsequently frozen at −80°C. Prior to DNA extraction, visible roots were removed. Afterwards, samples were treated with mechanical disruption using a microdismembrator (B. Braun Biotech International, Melsungen, Germany) for 3 min with 2000 rpm. Disrupted samples were dissolved in 600 μl sterile water. The DNA was directly extracted using the PowerSoil® DNA isolation kit (MO BIO Laboratories, Inc., Carlsbad, USA) as described by the manufacturer. The quality and purity of extracted DNA was tested with gel electrophoresis and NanoDrop ND-1000 spectrophotometer (Thermo Fisher Scientific, Wilmington, USA), respectively.

### Amplification of 16S rRNA gene

The V6–V8 region of the bacterial 16S rRNA gene was amplified with primers containing the Roche 454 pyrosequencing adaptors, keys as well as one unique MID per sample (underlined): F968 5′-CCATCTCATCCCTGCGTGTCTCCGAC-TCAG-(dN)_10_-AACGCGAAGAACCTTAC-3′ and R1401 5′-CCATCTCATCCCTGCGTGTCTCCGAC-TCAG-CGGTGTGTACAAGACCC-3′ (Nübel et al., 1996). The PCR (25 μl) contained following final concentrations: one-fold Phusion GC buffer (Thermo Scientific): 0.2 mM of each of the four deoxynucleoside triphosphates (Thermo Scientific), 0.2 μM of each primer, 0.04 U of Phusion high fidelity hot start DNA polymerase (Thermo Scientific), 5% DMSO and ~4 ng of the isolated soil DNA as template. The following thermal cycling scheme was used: initial denaturation at 98°C for 30 s, 30 cycles of denaturation at 98°C for 15 s, annealing at 53°C for 30 s, extension at 72°C for 30 s, followed by an additional extension step at 72°C for 2 min. Negative controls were performed using the reaction mixture without template. Genomic DNA of *Bacillus* was used as positive control. Three independent PCRs were performed per sample. Obtained PCR products were controlled for appropriate size and subsequently purified using the peqGOLD Gel Extraction Kit (Peqlab, Erlangen, Germany, now VWR) as recommended by the manufacturer. PCR products were quantified using the Quant-iT dsDNA HS assay kit and a Qubit fluorometer as recommended by the manufacturer (Thermo Scientific). Purified PCR products from the three independent PCRs were subsequently pooled in equal amounts. The Göttingen Genomics Laboratory determined the 16S rRNA gene sequences employing the Roche GS-FLX+ pyrosequencer with Titanium chemistry as recommended by the manufacturer (Roche, Mannheim, Germany).

### Processing and analysis of 454 pyrosequencing derived data

Pyrosequencing derived 16S rRNA gene data were preprocessed with QIIME version 1.8 (Caporaso et al., 2010). Preprocessing included the removal of short reads (<300 bp) as well as reads containing long homopolymer stretches (>8 bp) and too many primer mismatches (>3 bp) in the forward primer. Filtered data was subsequently denoised employing Acacia version 1.53b (Bragg et al., 2012). Remaining primer sequences were truncated employing cutadapt version 1.0 (Martin, 2011). Chimeric sequences were removed using USEARCH version 7.0.1090 (Edgar, 2010). For this purpose, sequences were first dereplicated in full-length mode and putative chimeras were initially removed using the UCHIME algorithm in *de novo* mode and subsequently in reference mode using the most recent SILVA database (SSURef 119 NR) as reference dataset (Edgar et al., 2011; Klindworth et al., 2013). Afterwards, processed sequences of all samples were joined and clustered in operational taxonomic units (OTUs) at 3% genetic dissimilarity according to Wemheuer et al. (2012) employing the UCLUST algorithm (Edgar, 2010). To determine taxonomy, a consensus sequence for each OTU was classified by BLAST alignment against the Silva SSURef 119 NR database (Camacho et al., 2009). Alpha diversity indices were calculated with QIIME as described by Wemheuer et al. (2014). Rarefaction curves were calculated in QIIME and subsequently interpolated in R (R Core Team, 2014) using the “drc” package [https://cran.r-project.org/web/packages/drc/]. The statistical analysis was performed in R.

### Isolation and enrichment of bacteria

Bacterial strains were obtained from root-associated soil samples. Soil samples were enriched for *Bacillus* strains, especially for Bt subspecies as described by Patel et al. (2011) using a modified glucose yeast extract salt (GYS) sporulation medium, which contained the following ingredients per liter: 1 g glucose, 2 g yeast extract, 2 g NH_4_(SO_4_)_2_, 0.06 g MnSO_4_, 0.4 g MgSO_4_·7H_2_O, 0.08 g CaCl_2_ and 5 g KH_2_PO_4_. Resulting colonies were picked with sterile toothpicks and inoculated in 96-deep well plates containing 2 ml two-fold concentrated lysogeny broth (TLB; Bone and Ellar, 1989). After overnight shaking at 30°C with 50 rpm (Orbitron S-000119510, Infors HT, Bottmingen, CHE), all colonies were stamped on lysogeny broth (LB; Sambrook and Russell, 2001) agar plates using a sterile 96 deep well plate replicator. The plates were incubated at 30 and 21°C for 24 h.

### Phenotypic analysis of isolates

Specific growth of cells was monitored after incubation at 30°C for 24 h using Bino Olympus SZX12, Olympus SC30 camera and the Olympus Cell Sense software. Different media were tested, nutrient rich liquid LB as well as modified solid nutrient limiting Simulated Xylem Medium (SXM). SXM was prepared according to (Dixon and Pegg, 1971) which contained following ingredients per liter: 2 g pectin, 4 g casein, 20 ml AspA (50x), 2 ml MgSO_4_ (1M), 1 ml trace elements (1000x; de Serres and Hollaender, 1982), and 20 g agar. AspA (50x) contained the following ingredients per liter: 300 g NaNO_3_; 26 g KCl and 76 g KH_2_PO_4_. Strains were tested for hemolytic activity on Columbia blood agar with Oxoid sheep-blood “plus” (Thermo Scientific). Hemolytic activity was monitored and measured after 48 h.

### Classification of isolated bacterial strains

Genomic DNA of each strain was extracted from overnight cultures grown in LB medium at 30°C with 150 rpm (New Brunswick Incubator Shaker Innova 2300, Neu-Isenburg, Germany) by using the MasterPure™ Complete DNA Purification Kit (Epicentre, Madison, USA) according to the manufacturer's instructions. The purified DNA served as template for multi-locus sequence typing (MLST) amplification by PCR according to Priest et al. (2004). Primers for seven house-keeping genes (*glpF, gmk, ilvD, pta, purH, pycA*, and *tpi*) are listed in Supplementary Table 1. The PCR mixture (50 μl) contained following final concentrations: one-fold OptiBuffer, 3 mM MgCl_2_, 0.04 U BIO-X-ACT™ short DNA polymerase (Bioline, London, UK), 1 mM deoxynucleoside triphosphates (Thermo Scientific), 0.4 μM of each primer, sterile water, and ~2 ng of bacterial DNA as template. Thirty cycles were conducted in a Mastercycler (Eppendorf, Hamburg, Germany). An initial denaturation at 98°C for 5 min was followed by 45 s denaturation at 98°C, 45 s annealing at 65°C, and 30 s primer extension at 72°C with a final step of 72°C for 5 min. PCR products were controlled for appropriate size and purified employing the QIAquick PCR purification kit (QIAGEN, Hilden, Germany) and eluted in 35 μl sterile water. Quantification of PCR products was performed via Nanodrop 1000 Spectrophometer (Thermo Scientific). The Göttingen Genomics Laboratory determined the sequences of the PCR products. For classification of the new isolates, all MLST genes from Bcsl representative genomes at time of analysis were considered. The corresponding sequences were obtained from GenBank hosted at the National Center for Biotechnology Information (http://www.ncbi.nlm.nih.gov/genome/genomes/486?). To exclude putative Ba isolates, all strains were tested for specific Ba virulence factors. Each MLST gene was aligned to determine the corresponding regions of each housekeeping gene and was trimmed to shortest shared region, resulting in orthologous sequences from 298 to 394 bp, as previously described (Jolley et al., 2004). Analysis was performed with concatenated DNA sequences of all seven gene loci. For construction of a phylogenetic tree, MEGA software 7.0.14 was used (Kumar et al., 2016). Sequence alignment was performed using the ClustalW algorithm and a phylogenetic tree was constructed by using the neighbor-joining method (Saitou and Nei, 1987). The robustness of the tree was evaluated by bootstrap analysis with 1000 resamplings.

### Whole genome sequencing and assembly

DNA extracted from isolated bacteria was subjected to whole-genome sequencing (Table 1) using the MiSeq sequencer (Illumina, San Diego, USA). For this purpose, Nextera_XT (Illumina, San Diego, USA) paired-end libraries (2 × 301 bp) were prepared according to the manufacturer's protocols. Resulting reads were quality-filtered with Trimmomatic 0.32 (Bolger et al., 2014) and evaluated using Fastqc (Bahabram Informatics, Babraham Institute; UK). Spades 3.5.0 (Bankevich et al., 2012) was used to assemble processed reads. For scaffolding, the resulting contigs were aligned to reference genomes Bt 407 (CP003889) and Bw KBAB4 (CP000902) using the Mauve Genome Alignment software (Darling et al., 2004). Sequencing results and genome characteristics are summarized in Table 1. Automatic annotation was carried out with the IMG-ER (Integrated Microbial Genomes-Expert Review) system (Markowitz et al., 2009) and with Prokka v1.9 (Seemann, 2014) using Bt 407 (Sheppard et al., 2013) as species reference and a comprehensive toxin protein database (including Cry, Cyt, Vip, Sip proteins). The Prokka pipeline was applied using gene calling by prodigal (Hyatt et al., 2010), rRNA genes and tRNA genes identification with RNAmmer 1.2 (Lagesen et al., 2007) and Aragorn (Laslett and Canback, 2004), respectively. Additionally, signal peptides were identified with SignalP 4.0 (Petersen et al., 2011) and non-coding RNA species with an Infernal 1.1 search against the Rfam database (Eddy, 2011).

**Table 1:** **Genome statistics of the 20 selected isolates**.

**Strain**	**Genome size [bp]**	**Number of contigs**	**Mean coverage**	**GC [%]**	**Total number of genes**	**Protein coding genes**	**RNA genes**	**CRISPR**	**GenBank accession**
Bt GOE1	5,359,363	30	214.5	35.1	5499	5389	109	0	LXLF00000000
Bt GOE2	5,373,416	43	122.1	35.1	5493	5383	110	0	LXLG00000000
Bt GOE3	5,347,504	60	146.1	35.1	5455	5340	114	0	LXLM00000000
Bt GOE4	6,008,382	63	110.9	34.8	6079	5967	111	0	LXLH00000000
Bt GOE5	5,772,687	123	123.7	35.1	5829	5736	92	0	LXLN00000000
Bt GOE6	5,857,591	187	75.3	35	6001	5926	74	0	LXLI00000000
Bt GOE7	5,976,466	91	124.2	35	5976	5898	77	1	LXLJ00000000
Bw GOE1	5,613,589	113	100.6	35.3	5742	5617	124	0	LXLK00000000
Bw GOE2	5,581,838	79	97.6	35.2	5722	5611	110	0	LXLO00000000
Bw GOE3	5,644,666	117	100.6	35.2	5787	5676	110	0	LXLP00000000
Bw GOE4	5,597,134	137	96.4	35.2	5733	5614	118	0	LXLQ00000000
Bw GOE5	5,589,822	151	110.4	35.3	5679	5564	114	0	LXLR00000000
Bw GOE6	5,632,050	118	98.1	35.2	5761	5643	117	0	LXLS00000000
Bw GOE7	5,772,687	126	185.2	35.2	5812	5699	112	0	LXLT00000000
Bw GOE8	5,851,749	156	113	35.2	5989	5882	106	0	LXLU00000000
Bw GOE9	5,846,892	144	142.2	35.2	5969	5885	83	0	LXLV00000000
Bw GOE10	5,823,784	143	78.1	35.2	5963	584	122	1	LXLW00000000
Bw GOE11	5,625,374	105	215.3	35.2	5776	5654	121	0	LXLX00000000
Bw GOE12	5,674,772	128	103.5	35.3	5805	5686	118	0	LXLY00000000
Bw GOE13	5,642,420	146	101.2	35.2	5763	5648	114	0	LXLL00000000

### Genome analysis of bacterial isolates

The genomes of all strains were screened for genes encoding Bt-specific toxins, such as crystal toxins, cytolytic toxins, vegetative insecticidal protein-toxins, and secreted insecticidal protein toxins (Cry, Cyt, Vip, and Sip toxins), and for candidate-genes for the production of antifungal secondary metabolites and chitinases. For identification of Cry-toxins, all protein sequences derived from the genome sequences were scanned using HMMSCAN implemented in HMMER v.3.1b2 (hmmer.org; Eddy, 1998) against a HMM-profile database. The profile database was generated from holotype Cry toxin sequences retrieved from the official BT Toxin Nomenclature website (http://www.btnomenclature.info/) and Uniprot (Bateman et al., 2015), respectively. To generate toxin-specific HMMs, all toxin sequences were grouped by Markov Cluster Algorithm v.12068 (Enright et al., 2002; van Dongen, 2007) according to the default options with an inflation value (−I) of 10.0. Representative members of each cluster were aligned by ClustalW v.1.83 (Thompson et al., 1994) and used as input to build the model database. After optimization of the models for sensitivity and specificity, the resulting HMMs were used for detection. Resulting group hits were further classified accordingly to the Cry Toxin Nomenclature. For the detection of Sip, Vip, and Cyt toxins, all protein sequences annotated accordingly from Uniprot (Bateman et al., 2015), National Centre for Biotechnology Information (NCBI, http://www.ncbi.nlm.nih.gov/), and the holotype-toxin list (http://www.btnomenclature.info/) were controlled for duplicates by 100% sequence identity. Only one representative was kept to create a BLASTp database, which was performed for each genome with an *e*-value of 1e^−50^. The analysis for secondary metabolites, i.e., polyketides, alkaloids, terpenes, phenanzines, microlides, and non-ribosomal antibiotic peptides was performed with antiSMASH 3.0 available at (https://antismash.secondarymetabolites.org/) (Weber et al., 2015). Chitinases were identified and compared to chitinase sequences of other Bcsl members. They were aligned using the ClustalW algorithm (Thompson et al., 1994) in MEGA7 (Kumar et al., 2016). To construct a phylogenetic tree, the Neighbor-Joining method (Saitou and Nei, 1987) and evolutionary distances were computed by the Maximum Composite Likelihood method (Tamura et al., 2004). InterPro (Mitchell et al., 2015) was used to group detected chitinases into chitinase families and for the prediction of domains, signatures, and active sites.

### Co-cultivation assay

To evaluate the antifungal potential of 20 phenotypically diverse *Bacillus* strains, co-cultivation assays with the two phytopathogens *V. dahliae* JR2 (Fradin et al., 2009) and *V. longisporum* 43 (Zeise and von Tiedemann, 2002) were conducted. All used strains are listed in Table 2. The insecticidal Bt subsp. *kurstaki* 2 and the Bt subsp. *israelensis* ONR60A strain were obtained from the *Bacillus* Genetic Stock Centre (BGSC; Columbus; USA) and served as reference strains. Three nematocidal strains (Bt MYBT18246, Bt Bt18247, and Bt Bt18679) and one insecticidal Bt strain (Btt) were used (Milutinovic et al., 2013; Masri et al., 2015). *Escherichia coli* DH5α served as negative control. Co-cultivation assays with two phytopathogenic fungi and selected *Bacillus* strains were performed using three different media. For standard bacterial cultivation, LB was used. The complex pectin containing SXM with limited nutrition tries to simulate the nutrient conditions which are available for a fungus in a plant. Due to the restriction of a synthetic medium it is limited in simulating the natural plant environment, where water, minerals, organic acids and ~500 μm amino acids are available in highly specific concentrations (Singh et al., 2010). The potato dextrose medium (PDM; Carl Roth GmbH, Karlsruhe, Germany with 5 g/L agar), a full medium containing potato starch and glucose, were used for optimal cultivation of *Verticillium* (Gams et al., 1998). Solid media were prepared containing 2% agar. Approximately 1 × 10^5^ harvested *Verticillium* conidiospores (Beckman Coulter Counter Size Analyzer, Krefeld, Germany) were homogeneously inoculated per plate using sterile glass beads (2.85–3.45 mm in diameter). A hole (diameter = 0.9 cm) was excised in the middle of the plate. This *in vitro* setting was designed to study *Bacillus/Verticillium* interactions under controlled conditions simulating the substrate supply of a plant and animal-associated habitat.

**Table 2:** **Organisms used in this study**.

**Abbreviation**	**Strain**	**Source**
4D2	*Bacillus thuringiensis* subsp. *kurstaki* 2	BGSC
4Q1	*Bacillus thuringiensis* subsp. *israelensis* ONR60A	BGSC
Bt GOE1	*Bacillus thuringiensis* isolate	This study, personal request
Bt GOE2	*Bacillus thuringiensis* isolate	This study, personal request
Bw GOE1	*Bacillus weihenstephanensis* isolate	This study, personal request
Bt GOE3	*Bacillus thuringiensis* isolate	This study, personal request
Bw GOE2	*Bacillus weihenstephanensis* isolate	This study, personal request
Bt GOE4	*Bacillus thuringiensis* isolate	This study, personal request
Bt GOE5	*Bacillus thuringiensis* isolate	This study, personal request
Bt GOE6	*Bacillus thuringiensis* isolate	This study, personal request
Bw GOE3	*Bacillus weihenstephanensis* isolate	This study, personal request
Bw GOE4	*Bacillus weihenstephanensis* isolate	This study, personal request
Bw GOE5	*Bacillus weihenstephanensis* isolate	This study, personal request
Bw GOE6	*Bacillus weihenstephanensis* isolate	This study, personal request
Bw GOE7	*Bacillus weihenstephanensis* isolate	This study, personal request
Bw GOE8	*Bacillus weihenstephanensis* isolate	This study, personal request
Bw GOE9	*Bacillus weihenstephanensis* isolate	This study, personal request
Bw GOE10	*Bacillus weihenstephanensis* isolate	This study, personal request
Bw GOE11	*Bacillus weihenstephanensis* isolate	This study, personal request
Bw GOE12	*Bacillus weihenstephanensis* isolate	This study, personal request
Bw GOE13	*Bacillus weihenstephanensis* isolate	This study, personal request
Bt GOE7	*Bacillus thuringiensis* isolate	This study, personal request
Bt MYBT18246	*Bacillus thuringiensis* MYBT18246	Available at Schulenburg lab^a^ (Masri et al., 2015)
Bt Bt18247	*Bacillus thuringiensis* Bt18247	Available at Schulenburg lab^a^ (Masri et al., 2015)
Bt Bt18679	*Bacillus thuringiensis* Bt18679	Available at Schulenburg lab^a^ (Masri et al., 2015)
Btt	*Bacillus thuringiensis* biovar *tenebrionis*	Available at Kurtz lab^b^ (Milutinovic et al., 2014)
DH5α	*Escherichia coli* DH5α (*fhuA2Δ(argF-lacZ)U169 phoA glnV44 Φ80Δ (lacZ)M15 gyrA96 recA1 relA1 endA1 thi-1 hsdR17)*	New England BioLabs, C2989K
*Vd* JR2	*Verticillium dahliae* JR2	Fradin et al., 2009
*Vl* 43	*Verticillium longisporum* 43	Zeise and von Tiedemann, 2002

*Supplementary information on organisms used in this study*.

a *Department of Evolutionary Ecology and Genetics, Zoological Institute, Christian-Albrechts-University of Kiel, Kiel, Germany*.

b *Animal Evolutionary Ecology Group, Institute for Evolution and Biodiversity, University of Münster, Münster, Germany*.

Bacteria were inoculated in 5 ml LB rotating with 120 rpm (New Brunswick Incubator Shaker Innova 2300, Neu-Isenburg, Germany) overnight at 37°C (Bt strains) or 25°C (Bw strains). Isolates were centrifuged for 2 min with 6000 rpm and washed with 2 ml sterile water. Afterwards, 5 ml of LB, PDM, or SXM were inoculated and adjusted to an OD_595_ of 0.1. Main PDM Bw cultures were inoculated to an OD_595_ of 0.01 and grown overnight at 25°C to an OD_595_ of 1. All other main cultures were grown at 30 or 25°C rotating with 120 rpm (New Brunswick Incubator Shaker Innova 2300, Neu-Isenburg, Germany) to an OD_595_ of 1. When the optical density was reached, 60 μl of the respective bacterial cultures were added into the hole in the plates. Plates were incubated at 25°C for 7 days. Zones of fungal growth inhibition were measured and quantified. Media without bacterial cells were used as positive controls for fungal growth. Each treatment was performed in technical triplicates and biological duplicates. To analyze possible differences between the isolates, a repeated measures ANOVA (Crawley, 2007) was conducted in R due to spatial pseudoreplication.

### Deposition of isolated strains and genome sequences

Sequence data were deposited in the Sequence Read Archive (SRA) of the NCBI under the accession number SRA401353. Genome sequence data were deposited in JGI and GenBank (Table 1). Strains are available on request (Table 2).

## Results and discussion

### Soil bacterial communities of tomato plants

Root-associated bacterial community composition and diversity were assessed by amplicon-based analyses of the V6-V8 region of the bacterial 16S rRNA gene. After quality filtering, denoising, and removal of potential chimeras and non-bacterial sequences, a total of 13,596 high-quality sequences were retrieved and used for further analyses. These sequences were grouped into 3994 OTUs. Calculated rarefaction curves (Supplementary Figure 1) as well as the mean coverage at species level (Table 3) revealed that the majority of bacterial community was recovered by the surveying effort. Richness (number of observed OTUs) and diversity (Shannon indices) for bacterial communities ranged from to 673 to 740.6 and 5.72 to 5.82, respectively (Table 3). All sequences could be classified below phylum level.

**Table 3:** **Diversity (represented by the Shannon index H') and richness (number of observed OTUs) of tomato-associated bacterial communities**.

**Sample**	**Richness**	**Diversity**	**Michaelis-Menten-Fit**	**Coverage**
Rep1	740.6	5.82	1006.46	0.74
Rep2	673.0	5.72	898.02	0.75
Rep3	740.3	5.81	1033.25	0.72

**Figure 1:**
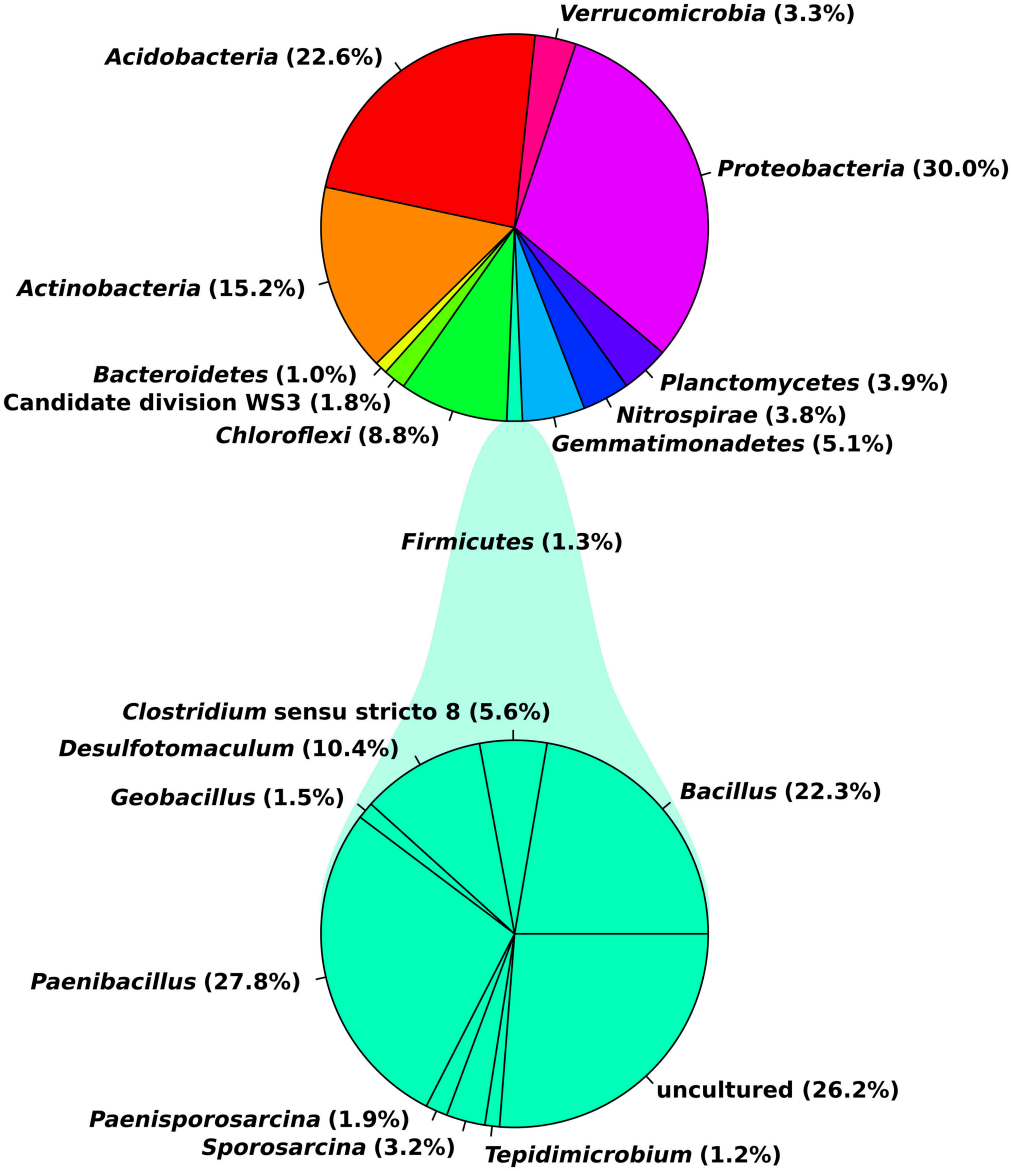
**General soil bacterial community composition at phylum level and for the ***Firmicutes*** at genus level**. Plots were generated in R.

Eleven abundant phyla (>1% of all sequences across all samples) were present in each soil sample and accounted for more than 96% of all bacterial sequences analyzed (Figure 1). *Proteobacteria* (30.0%), *Acidobacteria* (22.6%) and *Actinobacteria* (15.2%) were the most abundant (>1% abundance of all sequences) bacterial phyla. The *Proteobacteria* were mainly represented by *Beta*- and *Gammaproteobacteria*. Other prominent phyla were *Chloroflexi* (8.8%), *Verrucomicrobia* (3.3%) and *Firmicutes* (1.3%). Although their relative abundance is variable, these phyla have been previously found in different soils (Shange et al., 2012) and in the rhizosphere of different plant species (Romero et al., 2014; Pii et al., 2016). Li J. G. et al. (2014) investigated bacterial communities in roots and rhizosphere soils of healthy and diseased tomato plants and found that *Proteobacteria* was the most abundant phylum across all samples, followed by *Actinobacteria* and *Bacteroidetes*. This is in line with a study of Bulgarelli et al. (2015) on bacterial communities in bulk soil, rhizosphere, and roots of barley. In contrast to the above-mentioned findings, *Verrucomicrobia* (24%), *Acidobacteria* (23%) and *Proteobacteria* (17%) were the major taxonomic groups in rhizosphere bacterial communities of tomato (Romero et al., 2014).

In our study, observed genera included *Blastocatella* (2.6%), *Massilia* (1.64%) *Gaiella* (2.0%), *Nitrospira* (1.76%), *Paenibacillus* (0.34%), *Bacillus* (0.27%), *Bradyrhizobium* (0.17%), *Burkholderia* (0.04%), and *Pseudomonas* (0.006%; Supplementary Table 2). Most sequences of the *Firmicutes* were assigned to the two genera *Paenibacillus* (27.8%) and *Bacillus* (22.3%). Several of the observed genera including *Bacillus, Burkholderia*, and *Pseudomonas* are reported as the most significant phosphate-solubilizing bacteria (Bhattacharyya and Jha, 2012). Moreover, members of the genera *Bacillus, Paenibacillus, Bradyrhizobium, Pseudomonas*, and *Burkholderia* are known for their plant growth-promoting functions and/or their use as biocontrol agents against different phytopathogens and pests (Bhattacharyya and Jha, 2012; Glick, 2012). In a previous investigation on cultivable bacteria associated with tomato leaves, *Bacillus* (*Firmicutes*) showed strong *in vitro* antifungal activity against three important pathogens of tomato (*Botrytis cinerea, Fulvia fulva*, and *Alternaria solani*; Enya et al., 2007).

### Classification of bacterial isolates

The whole Bcsl taxonomy is recently discussed based on the different methods that are used for strain classification (Okinaka and Keim, 2016). Depending on the specific focus of the study, pathogenic properties or taxonomic features are used for classification resulting in an inconclusive taxonomy. We tried to address this question by performing MLST analysis as described by Priest et al. (2004) in combination with the analysis of phenotypic, biochemical and pathogenic characteristics. Our strain collection comprised 267 *Bacillus* strains. Twenty isolates with diverse colony morphologies were selected for further analysis.

**Figure 2:**
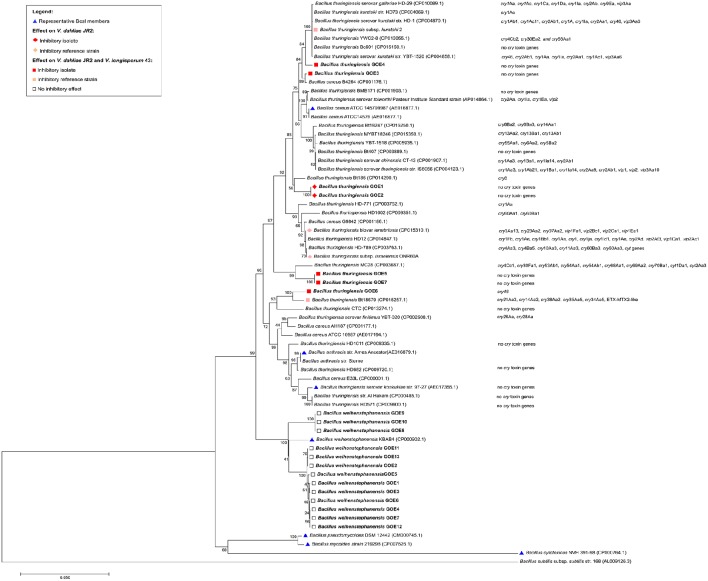
**Phylogenetic analysis of 20 Bcsl group isolates**. The 20 newly isolated strains are depicted in bold. (Blue triangle) Representatives of the Bcsl group species members. Strains which have been tested and showed an effect on *V. dahliae* JR2 or both *V. dahliae* JR2 and *V. longisporum* 43 are indicated by a diamond or square, respectively. Effects on *V. dahliae* JR2 (red diamond): inhibitory isolates, (beige diamond) inhibitory reference strains. Effect on *V. dahliae* JR2 and *V. longisporum* 43 (red square): inhibitory isolate, (beige square) inhibitory reference strain, (white square) no inhibitory effect. The phylogenetic analysis was performed using MEGA7 (Kumar et al., 2016), details are described in the Materials and Methods Section.

For the genome analysis of strains, the DNA was isolated and sequenced. Accession numbers and sequencing metadata are summarized in Table 1. The genome sizes ranged from 5.3 to 6.0 MB with a mean GC-content of 35%, which is typical for members of the Bcsl group (http://www.ncbi.nlm.nih.gov/genome/genomes/486?). The total number of genes varied from 5456 to 6080 and the number of identified RNA genes from 157 to 194. As the taxonomy of the Bcsl group is complex, the classical 16S rRNA phylogeny used for sequence based species assignments was ineffective for differentiation within this group. Based on MLST, 7 *B. thuringiensis* (Bt GOE1-7) and 13 *B. weihenstephanensis* (Bw GOE1-13) strains were identified (Table 1, Figure 2). None of the isolates clustered in close proximity to the human pathogen Ba or to any of the other known human pathogenic strains from the Bcsl species group. The analysis of similarity using the known Ba toxin genes or capsule genes such as *lef*, *cya, pagA*, and *capA*-*capC* showed that none of these genes are present in the new isolates. Additionally, there were no similarities to the four species-specific prophages (lambda01-lambda04), which are used for the PCR-based identification of Ba strains (Kolstø et al., 2009). All seven Bt isolates encode genes for the PlcR regulator with 100% sequence identity to Bt-reference sequences whereas no Bw isolate contained a *plcR* gene (Supplementary Table 3).

**Figure 3:**
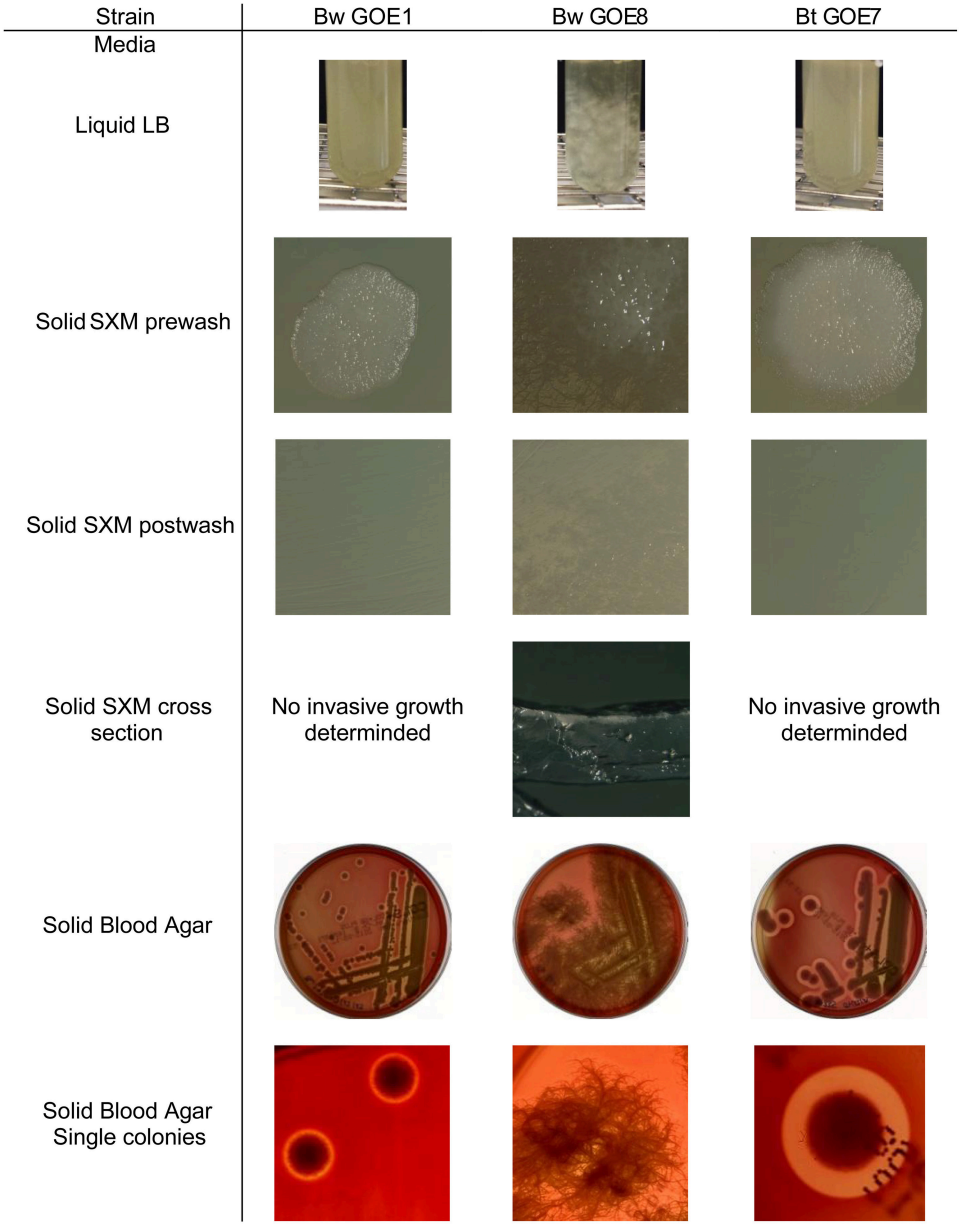
**Representative colony morphology phenotypes of tomato isolates**. The growth of three different strains, Bw GOE1, Bw GOE8, and Bt GOE7, was tested on several media. (Top row) Strains in liquid LB media, (2nd, 3rd, and 4th row) close up of single colonies on solid SXM media, pre/after scraping and washing with spatula and sterile water, and cross section of the agar. (5th and bottom row) Growth on solid blood agar and close up of single colonies. Strain abbreviations are explained in Table 2.

The morphology of Bcsl members was previously described in general as similar: the colony shape is irregular with undulate or curled margins (De Vos et al., 2009). Moreover, the colonies are flat to raised and opaque while Bm and Bp exhibit a mycoid to rhizoid growth with hairy-looking adherent colonies (De Vos et al., 2009). The morphological analysis of 20 isolates revealed two different growth-types in liquid as well as on solid media (Figure 3). The first growth type is represented by 7 Bt and 10 Bw isolates which formed circular to weakly irregular colonies with entire or undulate edges. The surface texture was ground glass to granular. The Bt isolates exhibited larger single colony sizes compared to Bw strains. These 17 strains were hemolytic positive on blood agar (Table 4, Figure 3). In liquid LB medium, the bacterial solutions were white yellowish and grew equally cloudy. In contrast, three Bw isolates (Bw GOE8-10) exhibited a Bm-like colony shape, with rhizoid growth on solid medium. The cells grew adherent and covered rapidly the whole agar plate as it is known for Bm which is in contrast to our MLST analysis (Figure 3; Di Franco et al., 2002). Moreover, the isolates displayed an invasive growth into the media plates (Figure 3). To the best of our knowledge, this has not been described previously for Bw strains. In addition, hemolytic activity could not be determined. In liquid medium, the strains produced an aggregation of clumps comparable to Bm (Di Franco et al., 2002). All Bw isolates showed mesophilic growth at 30°C instead of the described psychrophilic growth (Di Franco et al., 2002).

**Table 4:** **Hemolytic activity assay**.

**Bacterial Strain**	**Hemolytic activity on blood agar plates**
*E. coli* DH5α	–
4D2^*^	++
4Q1^*^	++
Bt GOE1	++
Bt GOE2	++
Bw GOE1	+
Bt GOE3	++
Bw GOE2	+
Bt GOE4	++
Bt GOE5	++
Bt GOE6	++
Bw GOE3	+
Bw GOE4	+
Bw GOE5	+
Bw GOE6	+
Bw GOE7	+
Bw GOE8	nd
Bw GOE9	nd
Bw GOE10	nd
Bw GOE11	+
Bw GOE12	+
Bw GOE13	+
Bt GOE7	++
Bt MYBT18246^**^	–
Bt Bt18247^**^	+
Bt Bt18679^**^	++
Btt^*^	++

Symbols refer to the radius of the zone of clearance (mm): –, absence of zone; presence of zone of clearance: + = 0.5–2 mm; ++ ≥ 2 mm;

* insecticidal strains;

** *nematocidal strains; nd, not determined due to growth structure*.

To determine the pathogenic properties of the isolates, we screened for virulence factors specific for the Bt species group (Cry, Cyt Sip, and Vip toxins). Within the MLST tree (Figure 2), the Bt isolates clustered with *B. thuringiensis* reference strains, with present or absent Cry toxins. In contrast, all Bw isolates clustered with *B. weihenstephanensis* references (Figure 2). The strains Bt Bc601, Bt BMB171, Bt CTC, Bt HD1011, Bt HD682, Bt str. Al Hakam, and Bt HD571 contained no homologs to any known Cry, Cyt, or Vip toxins (Figure 2). Notably, even in the type strain Bt serovar *konkukian* str. 97-27, which was isolated from a necrotic human wound, no full-length hits to known Cry, Cyt, or Vip toxins were identified (Han et al., 2006). Only Bt GOE6 contained a gene with similarity to Cry46. In all other isolates, no homologs to any known Cry, Cyt, Sip, or Vip toxins were detected using BLASTp and HMM models. Moreover, proteins sharing a domain with Cry6 and Cry22 were detected in all Bt and Bw isolates. Modeling and detection of known as well as for new Cry toxins is not trivial based on their variable structures. Cry toxins can be sub-grouped into three classes: Three-domain toxins, Bin-toxins and Mtx-toxins (de Maagd et al., 2003). Additionally, some Cry toxins are phylogenetically unrelated and unique such as Cry6, Cry22, Cry34, Cry37, Cry55, and Cry46 (Palma et al., 2014). It is possible that our strains encode novel Cry toxins. The proteins sharing a domain with Cry6 and Cry22 and might represent new toxin candidates. However, Cry toxins are often encoded on mobile elements located on plasmids (González et al., 1982). Cry toxin-encoding Bt plasmids may be instable and thus can get lost within cultivation (Masri et al., 2015; Sheppard et al., 2016).

The inconsistent combination of taxonomic relevant features supports the hypothesis of Okinaka and Keim (2016) that the Bcsl species group is complex and that exclusive phenotypic and biochemical characterization may be misleading. The authors suggest that the whole Bcsl complex should be considered as a single species. Investigation of only the phenotypic characteristics of the strains would lead to a mis-classification of Bt isolates, as no known Cry toxins were detected. Moreover, the invasive growing Bw strains would be mis-classified to the species Bm or Bp. However, there are examples for the challenging taxonomic classification of Bcsl members. Bt serovar, *navarrensis, bolivia*, and *vazensis* have been classified as Bt based on their ability to produce Cry toxins. They showed the typical psychrotolerant growth and encode the *cspA* gene signature, which is species specific for Bw (Soufiane and Côté, 2009). We classified our isolates as 7 Bt and 10 Bw based on our MLST study combined with phenotypic features (Figure 2). The three special Bw isolates (Bw GOE8-Bw GOE10) combined several morphological as well as genomic characteristics of the species Bm, Ba, and Bw indicating that they might represent a distinct subspecies within the species Bw (Figure 2). In conclusion, our findings indicate that a combination of MLST analysis and phenotypic, biochemical, and pathogenic classification is the only possibility to distinguish between members of the Bcsl complex.

### *In vitro* antagonistic properties of root-associated *Bacilli* toward *Verticillium*

**Figure 4:**
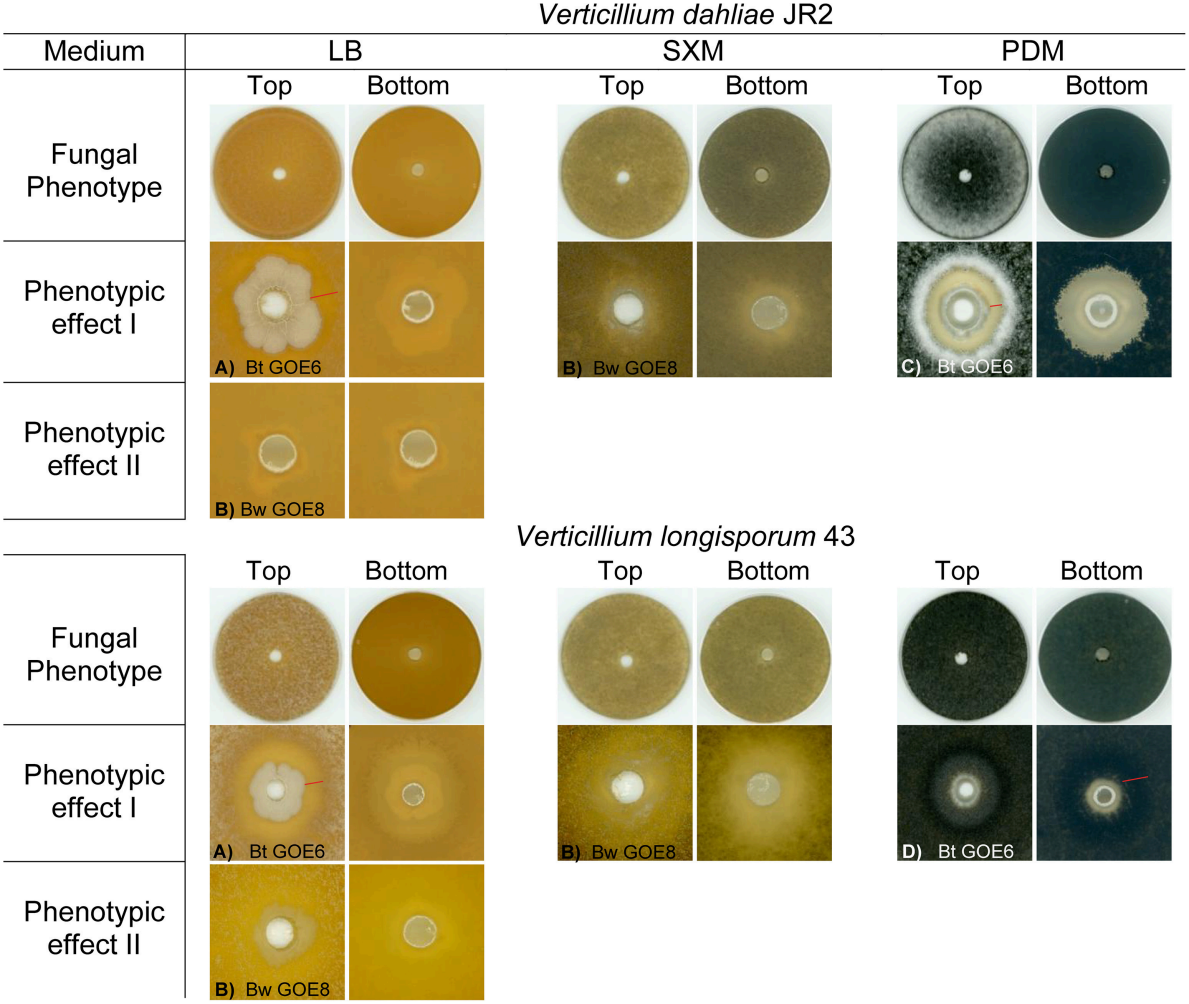
**Phenotypic effects of selected bacterial strains against ***Verticillia*****. Fungal phenotypes without bacteria are displayed in the 1st and 4th row. Specific interaction effects of *V. dahliae* JR2 or *V. longisporum* 43 and antagonistic bacterial strains on different media are depicted: **(A)** Bacteria produce biofilm with or without inhibition of the fungus, **(B)** Bacteria produce hyphae-like structures where the growth of the fungus is reduced, **(C)** White air mycelia is build by the fungus around the bacteria which inhibits *V. dahliae* JR2, **(D)** *V. longisporum* 43 showed a strong melanization surrounding the bacterial application site visible as a dark ring. Inhibition zone and melanization zone of fungi are depicted by a red bar. Strain abbreviations are explained in Table 2.

**Figure 5:**
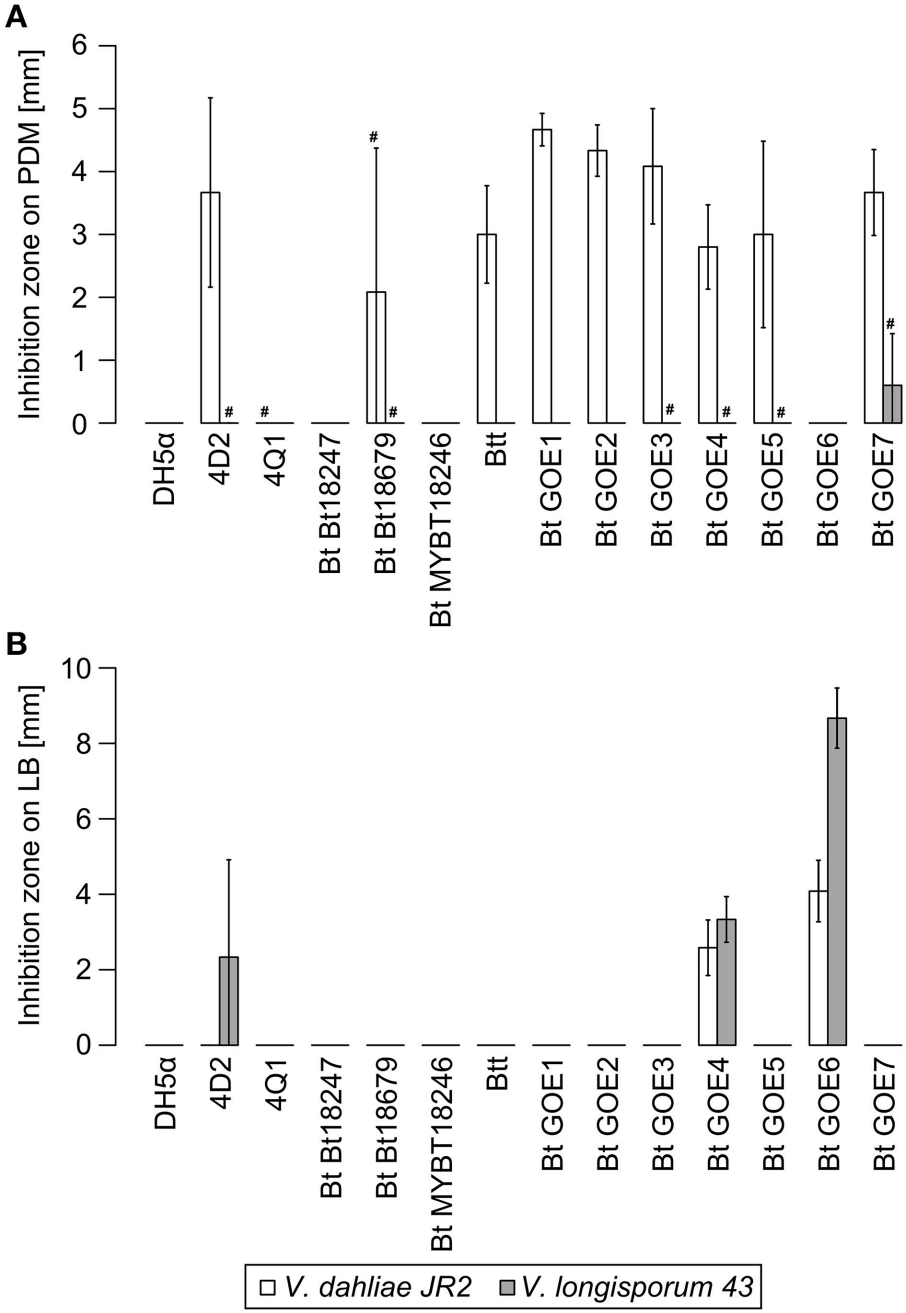
**Inhibition of ***V. dahliae*** JR2 and ***V. longisporum*** 43 by ***Bacillus*** isolates**. Mean inhibitory effects of all tested strains are displayed in [mm]: **(A)** Effect on PDM medium, **(B)** Effect on LB. *E. coli* DH5α served as negative control. Variation bars on the histogram corresponds to standard deviation. # indicate strains where a phenotypic effect was observed, but no measurable inhibition was quantified due to study limitations. Plots were generated in R. Strain abbreviations are explained in Table 2.

We further investigated the antagonistic potential of new isolated *Bacilli* strains against two phytopathogenic *Verticillium* species with different host ranges. In total, 20 *Bacillus* isolates and 6 Bt reference strains were tested for their ability to suppress the haploid tomato pathogen *V. dahliae* JR2 (Fradin et al., 2009) or the diploid rapeseed pathogen *V. longisporum* 43 (Zeise and von Tiedemann, 2002; Tran et al., 2013) on three different media (Table 2). Not all Bt reference strains were able to suppress *V. dahliae* JR2 and showed a broad range of different interaction effects against *V. longisporum* 43 indicating that antifungal activity is a property of a particular Bt strain and not of the whole species (Supplementary Table 4, Supplementary Figures 2, 3). The co-cultivation assays of *Bacillus* isolates revealed specific phenotypic effects for the antagonistic bacteria as well as for the phytopathogens (Figure 4). The different bacterial isolates varied in their ability to inhibit the mycelia growth of either *V. dahliae* JR2 or *V. longisporum* 43. A significant *in vitro* antagonism was observed for all Bt isolates against *V. dahliae* JR2 on PDM, excluding Bt GOE6 compared to the tested Bt control strains (Bt Bt18247, Bt MYBT18246, 4Q1) which exhibited no inhibitory effect (Figure 5A, Supplementary Table 5). A clear inhibition zone without *Verticillium* mycelium, sometimes with a slight formation of microsclerotia, was detected (Supplementary Figure 2). Additionally, *V. dahliae* JR2 showed an altered phenotype compared to the fungal control by the building of a strong white air mycelium around the bacteria (Figure 4C). The antagonistic effect of Bt varied but was consistent in all biological replicates and had a mean ranging from 1.5 to 8.6 mm (Figure 5). In contrast to all other tested Bt isolates, which exhibited an antagonistic effect on PDM, isolate Bt GOE6 showed a significant antagonism against *V. dahliae* JR2 only on LB (Figure 4A, Figure 5, Supplementary Table 5). Notably, none of the strains belonging to Bw showed an inhibitory effect on *V. dahliae* JR2 or *V. longisporum* 43 (Supplementary Table 4). In comparison, a weaker antagonistic effect of bacteria on *V. longisporum* 43 was recorded. All strains which had an effect on *V. dahliae* JR2 lead to an altered phenotype of *V. longisporum* 43 on PDM. The bacteria induced a stronger melanization in *V. longisporum* 43, which could be observed on the back of the plate as a black ring around the bacteria (Figure 4D). Only for strain Bt GOE7 an inhibitory effect with high variance in replicates was observed compared to all other tested strains (Figure 5, Supplementary Table 5). On LB, Bt GOE4 and Bt GOE6 exhibited a significant inhibitory effect against *V. longisporum* 43 compared to all other tested strains (Figure 5, Supplementary Table 5). Additionally, 4D2 showed an inhibitory effect aginst *V. longisporum* 43 as well but with an increased variance in biological treatments. Moreover, Bt GOE6 had a significant higher inhibitory effect against *V. longisporum* 43 compared to 4D2 and Bt GOE4. Significant inhibitory effects against both phytopathogens, *V. dahliae* JR2 and *V. longisporum* 43, were only observed for Bt GOE4 and Bt GOE6 on LB (Figure 5, Supplementary Table 5). However, two additional phenotypic effects of bacteria were observed: (a) biofilm production of bacteria with varying inhibition effects on fungi and (b) building of hyphae-like growth structures of bacteria leading to a suppression or growth reduction of both phytopathogens. Effect (a) was detected for all Bt isolates, which build moderate biofilms. Bw isolates conferred only a weak biofilm production with no suppression effect, excluding Bw GOE8-GOE10. Effect (b) was observed for isolates Bw GOE8-GOE10 on LB and SXM (Figure 4B, Supplementary Table 4). The hyphae-like structures or rhizoid growth enables the bacterium to invade the same habitat as *Verticillium*. This might strengthen the competitive access of *Bacilli* for nutrients, leading to suppression or reduction of fungal growth to different extents. As the isolated Bt strains suppressed *V. dahliae* JR2 growth, this indicates that tomato-associated Bt strains are able to inhibit phytopathogens. In contrast, the effect on the foreign plant pathogen *V. longisporum* 43 was in general weaker or even not detectable. We assume that different host-range specificities play an important role in *Verticillium* species, resulting in two different life strategies. *Verticillium dahliae* is haploid and comprises strains with a broad host range (Inderbitzin et al., 2011) whereas *V. longisporum* is amphidiploid (hybrid of *V. dahliae* and an unknown haploid *Verticillium* species) with narrow host range infecting mainly *Brassicacae* (Depotter et al., 2016). This could lead to long-term adaptation of *V. longisporum* resulting in an increased fitness, as *V. longisporum* has to persist in soil waiting for its specific host plant. *Verticillium dahliae* is able to colonize a broad range of host plants and thus has to cope with different environmental conditions. We suggest that this results in a reduced adaptation potential for the different types of host-residing pathogens. An opportunity to enter the next potential host are spores or the formation of resting structures, such as microsclerotia, which are formed under stress conditions and can easily spread by wind or survive in the ground.

On SXM, only minor and inconsistent effects were observed around the bacterial application site and not further quantified. Instead of an inhibition, a reduced formation of fungal mycelium was observed (Supplementary Figures 2, 3). Nonetheless, the ability of a bacterial strain to inhibit fungal growth differed depending on the medium. Using different phytopathogenic interaction partner had also an impact on phenotypic effects of bacteria (Supplementary Table 4). Generally, the inhibitory effect on *V. dahliae* JR2 was always more prominent than the effect on *V. longisporum* 43 (Figure 4, Supplementary Figures 2, 3). In particular, on PDM, the fungus is able to sense the bacteria or antifungal substances. This leads to a strong physiological reaction of *V. dahliae* JR2 resulting in an increased production of air-mycelium in distance to the bacterial application site. Only in individual cases, a reduced number of microsclerotia was detected (Supplementary Figure 2; Bt GOE5, Bt GOE7). For *V. longisporum* 43, an increased production of microsclerotia was observed indicating an induced stress response by bacteria. However, SXM is also a rich medium containing the complex heteropolysaccharide pectin, which is more difficult to use for bacteria. We suggest that this reduce or enhance the growth efficiency of bacteria or fungi, respectively. In contrast, LB medium is favored by bacteria, which could explain the biofilm production, whereas both fungi built minor mycelium (Figure 4).

### Secondary metabolites of root-associated *Bacilli*

The secondary metabolite gene prediction tool (AntiSMASH 3.0; Weber et al., 2015) was used to perform a comparative genome analysis and identify candidates contributing to the observed antifungal effects. In total, 304 gene clusters including 101 NRPS gene clusters and 203 RiPP gene clusters were identified (Table 5). In each genome, secondary metabolite gene clusters were detected, including genes assigned to the production of siderophores and terpenes. The gene clusters assigned to siderophore synthesis showed similarity (80–83%, BGC0000942) to a petrobactin biosynthetic gene cluster and a bacillibactin cluster (38–46%, BGC0000309.1). Both are catecholate siderophores, which are produced under iron-limited conditions, and present in Ba and other Bcsl members. Li B. et al. (2014) reported that the transcription of bacillibactin genes was upregulated when strains were confronted with fungal pathogens. Bacillibactin-deficient mutants exhibited no or reduced antagonistic effects. Although the suppression could not be observed in each treatment, the findings indicate that bacillibactin plays a supporting role in the suppression of the two different *Verticillium* species (Li B. et al., 2014).

**Table 5:** **Identified secondary metabolite gene clusters of bacterial strains**.

**Identified secondary metabolite gene cluster**	**NRPS**		RiPPs^*^						**Terpene**	**Others**	**Total**
	**Siderophore**	**Not further categorized**	**Bacteriocins**	**Microcins**	**Lanthipeptide**	**Lassopeptide**	**Linaridin**	**Ladderane**			
	**(Petrobactin, Bacillibactin)**										
**BACTERIAL STRAINS**											
Bt GOE1	2	3	3	1	1	–	–	–	1	1	12
Bt GOE2	2	4	3	1	1	–	–	–	–	1	12
Bw GOE1	2	1	2	1	–	1	–	–	1	–	8
Bt GOE3	2	2	3	1	–	–	–	–	1	1	10
Bw GOE2	2	1	2	1	–	1	–	–	1	–	8
Bt GOE4	2	3	4	1	4	–	–	–	1	2	17
Bt GOE5	2	3	5	1	–	1	–	–	1	1	14
Bt GOE6	2	3	3	1	–	–	–	–	1	1	11
Bw GOE3	2	1	2	1	–	1	–	–	1	–	8
Bw GOE4	2	1	2	1	–	1	–	1	1	–	9
Bw GOE5	2	1	4	1	–	1	–	–	1	–	10
Bw GOE6	2	1	2	1	–	1	–	–	1	–	8
Bw GOE7	2	1	2	1	–	1	–	1	1	–	9
Bw GOE8	2	1	3	1	1	2	–	–	1	1	12
Bw GOE9	2	1	3	1	1	1	–	–	1	1	11
Bw GOE10	2	1	3	1	1	2	–	–	1	1	12
Bw GOE11	2	1	3	1	–	1	–	1	1	–	10
Bw GOE12	2	1	2	1	–	1	–	1	1	–	9
Bw GOE13	2	1	3	1	–	1	–	1	1	–	10
Bt GOE7	2	2	4	1	–	1	1	–	1	2	14
Bt MYBT18246	2	2	6	8	1	–	–	–	1	1	21
Bt Bt18247	2	2	5	–	–	–	–	–	1	1	11
Bt Bt18679	2	3	4	–	–	–	–	–	1	–	10
Btt	2	4	3	–	–	–	–	–	1	1	11
Bt serovar *kurstaki* HD-1	2	5	3	8	1	–	–	–	1	1	21
Bw KBAB4	2	1	3	8	–	–	–	–	1	1	16

* *(RiPPs): Ribosomally synthesized and post-translationally modified peptides*.

**Figure 6:**
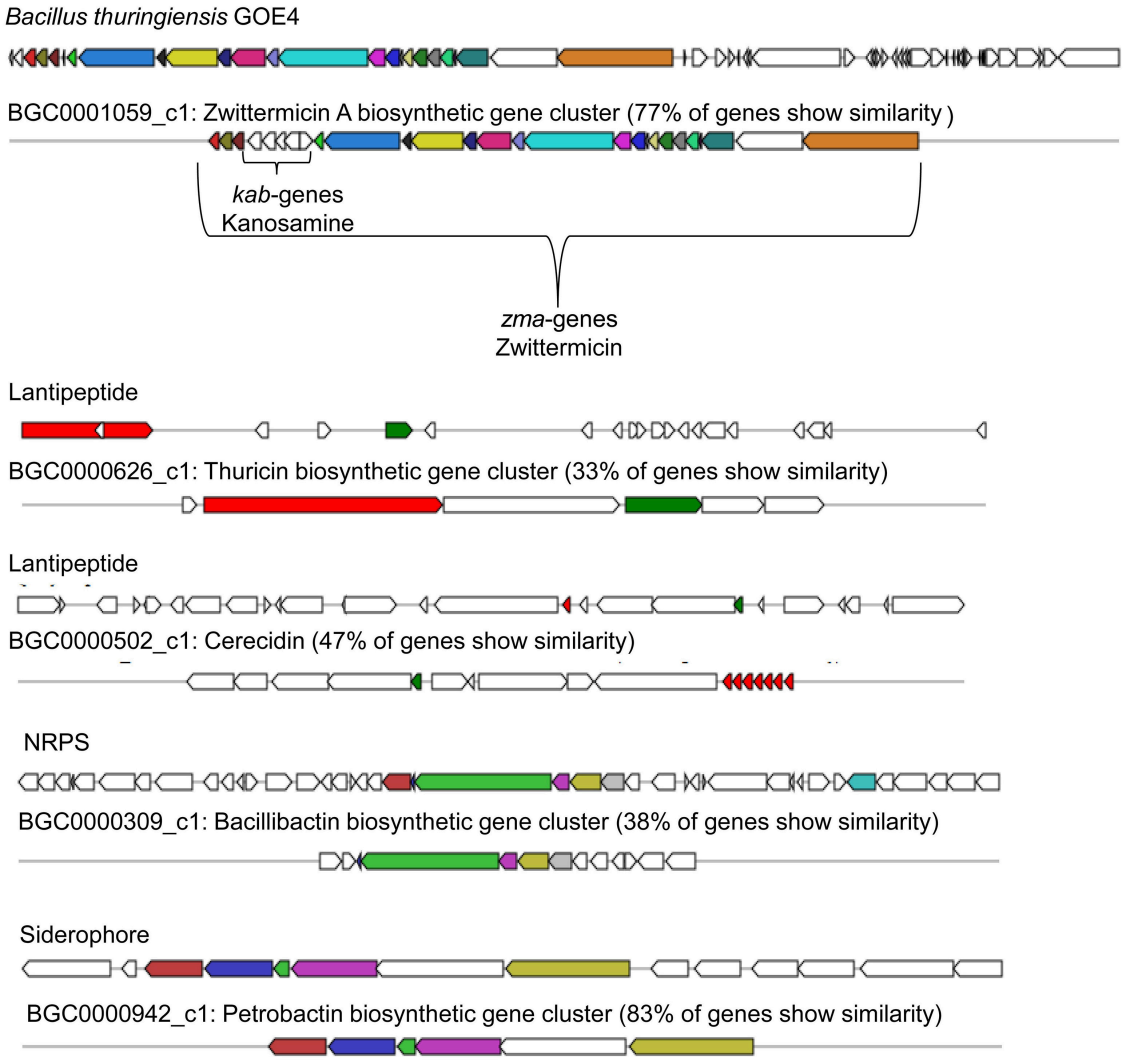
**Biosynthetic gene clusters of Bt GOE4**. The analysis was performed with AntiSMASH3.0 (Weber et al., 2015). Same colored genes display similarity to published and known gene clusters. A minimum of 30% cutoff was set for visualization of gene clusters.

It was suggested that petrobactin could contribute to virulence of Ba (Lee et al., 2011), but antifungal activity was not discussed. A number of plants including tomato are known to produce terpenes for deterring or attracting herbivores, parasites, and predators (Lange et al., 2000; Martin et al., 2003). Terpene assigned gene clusters were identified in all strains and shared only low similarity (11–17%, BGC0000916.1) with molybdenum cofactor biosynthesis genes. Bt GOE4 showed the highest number of detected secondary metabolites and antimicrobial peptides with similarity to known gene clusters with antifungal activity (Figure 6). One gene cluster putatively producing the antifungal zwittermicin A (BGC0001059) was detected. It belongs to the class of antibiotics of type I PKS gene clusters, which is known to suppress plant diseases (Handelsman et al., 1990). Zwittermicin A is a linear aminopolyol, which was first identified in *B. cereus* UW85 (Silo-Suh et al., 1994). The cluster was only identified in Bt GOE4 and in the reference genome Bt serovar *kurstaki* HD-1. In Bt GOE4, the cluster differs by lacking the five *kab* (*kabR; kabA-kabD*) genes, which are important for the kanosamine production. Kanosamine is not important for the synthesis of zwittermicin A, but it is also known to exhibit fungicidal activities (Janiak and Milewski, 2001). In these strains, a thuricin-like cluster (66% similarity, BGC0000626.1) and a thuricin H biosynthetic gene cluster (90% similarity, BGC0000600.1) were detected. Thuricin H is described as member of a small subclass of bacteriocins, which act on related bacterial strains and not on fungi (Mathur et al., 2015). A cerecidin cluster was detected (47%, BGC0000502) in Bt GOE4. Cerecidin is a novel antibiotic with high activity against a broad range of Gram-positive bacteria (Wang et al., 2014). In addition, a putative paenilamicin (BGC0001033.1) producing type I PKS gene cluster was exclusively identified in Bt18679 and Bw KBAB4 with shared similarity of 35%. Paenilamicins are known to act antibacterial and antifungal (Müller et al., 2014). As this gene cluster was not detected in all suppressing isolates, it is not exclusively the paenilamicins which lead to the inhibitory effect in our experiments.

Gene clusters encoding ribosomally synthesized and post-translationally modified peptides (RiPPs) such as bacteriocins, microcins, lanthipeptides, lassopeptides, ladderane, and linaride were identified as well, but could not be further characterized due to weak similarity to known clusters (Table 5). Only in case of the microcin bacitracin (BGC0000310.1), genes with similarities of 44% have been identified in the strains Bt subsp. *kurstaki* HD-1 and Bw KBAB4. Bacitracin is not known for a fungicidal effect, but rather for the effect on spore size and crystal production (García-Patrone, 1985). Gene clusters with the potential to encode antifungal compounds, such as zwittermicin A, are present in some strains. Remarkably, the genes are not shared by all isolates acting as fungal antagonists. In contrast, bacillibactin producing gene clusters were identified in each isolate independently of the ability to suppress the growth of *V. dahliae* JR2 or *V. longisporum* 43. We think that NRPS/PKS synthesized secondary metabolites or RiPPS could contribute to the observed growth inhibition. We strongly suggest that different complex mechanisms and substances act in combination and are important for an efficient suppression of the fungi investigated.

### Chitinases of root-associated *Bacilli*

**Figure 7:**
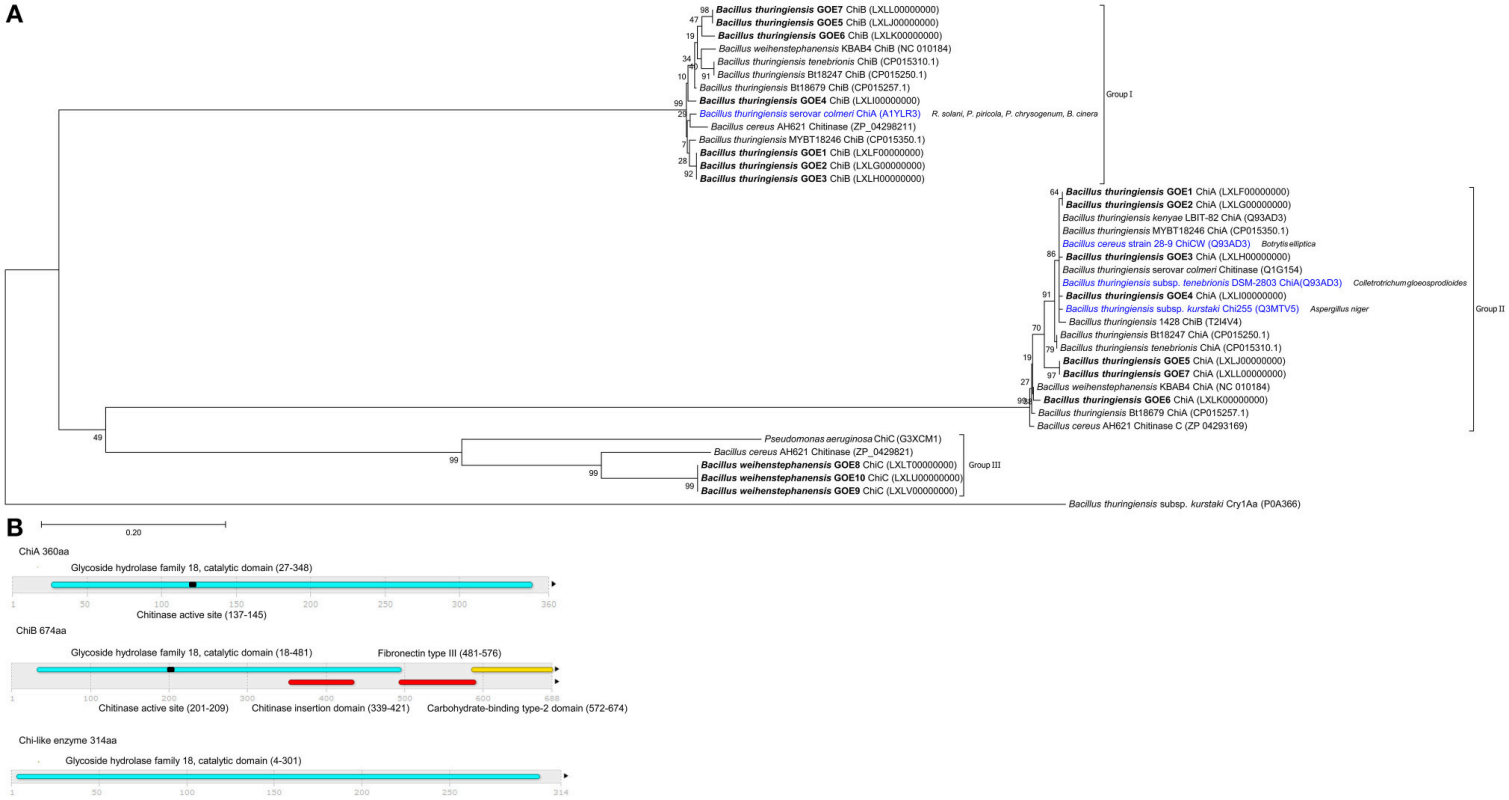
**Analysis and comparison of chitinases encoded by the genomes of the isolates. (A)** Phylogenetic tree of all identified chitinases (bold black) compared to chitinases of reference strains, with known antifungal activity (blue) target organism is depicted in brackets. The phylogenetic analysis was performed using in MEGA7 (Kumar et al., 2016), details are described in the Materials and Methods Section. **(B)** Domain structures of three identified chitinase types. InterPro (Mitchell et al., 2015) was used for functional analysis of chitinases, classification into families and for the prediction of domains and active sites. Identified structures are colored: (blue) glycoside hydrolase family 18 with catalytic domain, (red) chitinase insertion domain, fibronectin type III domain, (yellow) carbohydrate-binding type-2 domain, (black) chitinases active site.

To elucidate the second class of fungicidal substances, we focused on chitinases as the mycolytic activity of many *Bacilli* is known (Swiontek Brzezinska et al., 2014). Genome analysis revealed the presence of three different classes of chitinases shared by the genomes of the strains with antifungal activities. Notably, genes encoding a chitinolytic polypeptide with 674 (ChiA) and 360 (ChiB) amino acids were identified in all inhibiting isolates (Table 6, Figure 7) but not in Bw isolates, with exception of the invasive-growing Bw strains (Bw GOE8-10). These isolates contained a gene encoding a chitinase-like enzyme of 314 amino acids. Comparison of the amino acid sequences of all detected chitinases with reference chitinases with known antifungal activity lead to a classification of three chitinase groups: Group I: ChiB; Group II: ChiA and Group III: ChiC (Figure 7B). Identified ChiA chitinases showed sequence similarity to known mycolytic chitinases of *Bt s*ubsp. *tenebrionis* DSM-2803 (de la Fuente-Salcido et al., 2016), *B. cereus* strain 28-9 (Huang et al., 2005) and Bt subsp. *kurstaki* (Driss et al., 2005; Figure 7A). They grouped into glycoside hydrolase family 18 with a catalytic domain and an active site. ChiB chitinases clustered to reference chitinases, including ChiB of Bc AH621 and the mycolytic chitinase chiA of Bt serovar *colmeri* (Liu et al., 2010). They also grouped into glycoside hydrolase family 18, but in addition to the catalytic domain and the active site, the enzymes contained a chitin insertion domain and a carbohydrate-binding type2 domain, which are separated by a fibronectin type III domain.

**Table 6:** **Identified chitinases in bacterial strains**.

**Bacterial strain**	**Chtitinase**	**Length in bp**	**Length in aa**
Bt GOE1	ChiA	1083	360
Bt GOE1	ChiB	2025	674
Bt GOE2	ChiA	1083	360
Bt GOE2	ChiB	2025	674
Bt GOE3	ChiA	1083	360
Bt GOE3	ChiB	2025	674
Bt GOE4	ChiA	1083	360
Bt GOE4	ChiB	2025	674
Bt GOE5	ChiA	1083	360
Bt GOE5	ChiB	2025	674
Bt GOE6	ChiA	1083	360
Bt GOE6	ChiB	2025	674
Bw GOE8	ChiC-like	945	314
Bw GOE9	ChiC-like	945	314
Bw GOE10	ChiC-like	945	314
Bt GOE7	ChiA	1083	360
Bt GOE7	ChiB	2025	674
Bt MYBT18246	ChiA	1083	360
Bt MYBT18246	ChiB	2025	674
Bt Bt18247	ChiA	1083	360
Bt Bt18247	ChiB	2025	674
Bt Bt18679	ChiA	1083	360
Bt Bt18679	ChiB	2025	674
Btt	ChiA	1083	360
Btt	ChiB	2025	674
Bw KBAB4 ChiA	ChiA	1083	360
Bw KBAB4 ChiB	ChiB	2025	674
*Pseudomonas aeruginosa*	ChiC	1463	483
Bt serovar *colmeri* strain 15A3	ChiA	1083	360
Bt serovar *colmeri* strain 15A3 Chitinase	ChiB	2076	688
Bt serovar *kurstaki* Chi255	Chi255	2710	376
Bc strain 28-9 Chitinase CW	ChiCW	2450	674
Btt DSM-2803	ChiA	2331	676
Bt *kenyae* LBIT-82	ChiA	2331	676
Bt strain SBS-Bt5	ChiB	2331	676
Bc AH621	ChiC	912	303
Bc AH621	ChiB	2067	688
Bc AH621	ChiA	1083	360

Our experiments demonstrate that the classification of ChiA from Bt serovar *colmeri* should be reconsidered, as the deduced amino acid size of the protein and domain analysis revealed that this chitinase belongs to the ChiB group of chitinases (Group I). The chitinases of the invasive Bw strains were also classified as glycoside hydrolase family 18, containing a catalytic domain. They were most similar to a chitinase of *B. cereus* AH621 and *Pseudomonas aeruginosa* (Figure 7). In contrast, the Bw chitinases harbored no chitinase insertion domain, fibronectin type III domain, carbohydrate-binding type-2 domain or a chitinase active site. We suggest that we identified potential mycolytic chitinases in our Bt isolates whereas we can only hypothesize which of the identified groups of chitinases (chiA or chiB) are able to suppress *Verticillium*. Strain Bt GOE6 exhibited an antifungal effect on LB only but not on PDM. In addition, the genomes of the Bt control strains MYBT18246 and Bt18247 encode two chitinases but showed no effect on the fungal pathogens at all. These results suggest that the growth period and abiotic factors such as pH, temperature, and presence of metal ions, carbon, or nitrogen-sources influence the expression and/or the activity of chitinases. The identified chitinase in the invasive Bw strains exhibited less similarities to known sequences and thus could be a new type of chitinase. To our knowledge, this is the first time that several Bt isolates comprising multiple chitinases, showed the potential to inhibit *V. dahliae in vitro*. As chitinases are an effective biocontrol substance against a number of phytopathogenic fungi (Bhattacharya et al., 2007), future *in planta* experiments are needed to elucidate the full potential of chitinases of Bt isolates.

## Conclusion

In the present study, we evaluated the antifungal potential of 20 phenotypically diverse *Bacillus* isolates toward *V. dahliae* and *V. longisporum*. A classification of new isolates based on a combination of morphological, pathogenic, and taxonomic properties revealed 7 *B. thuringiensis* and 13 *B. weihenstephanensis* strains. The dual cultivation assays showed a correlation between taxonomy and antagonistic activities. All *B. thuringiensis* strains exhibited an *in vitro* antifungal effect against *V. dahliae* while only limited antagonism was observed against *V. longisporum*. Additionally, three *B. weihenstephanensis* isolates showing an invasive growth-type competed with both phytopathogenic fungi. The relation of the rhizoid growth and the mechanism of competition of *B. weihenstephanensis* strains have not been described previously and thus represent a fascinating new research topic.

The genome analysis of the 20 *Bacillus* strains revealed that strains with antifungal activity shared genes assigned to bacillibactin production and mycolytic chitinases, which are thus the most promising candidates for encoding the antifungal effect.

The hereby produced genomic and physiological data provide an excellent foundation for the identification, purification, and characterization of the active antifungal substances of Bt. Nonetheless, future *in planta* experiments are necessary to determine the efficacy of these strains in controlling plant pathogens.

## Author contributions

The study was designed and conceived by HL, RH, RD, and JH. Soil bacterial communities were analyzed by JH and FW. Bacterial isolation, PCR, and bacterial identification were performed by AK, JH, EB, and HL. Genome sequencing, data processing, and genome assembly were done by JH and AP. Genome annotation, data submission, and data interpretation were performed by JH, SDV, and HL. Antagonistic assays were undertaken by AK, RH, and JH. Data Interpretation and manuscript preparation were done by JH, FW, RH, and HL. The whole project was supervised by RH, SB-S, GB, KN, RD, and HL. All authors interpreted the results, read and approved the final manuscript.

## Funding

We thank the German Research Foundation (DFG-SPP1399, Grants LI 1690/2-1 and DFG BR1502/15-1) for financial support.

### Conflict of interest statement

The authors declare that the research was conducted in the absence of any commercial or financial relationships that could be construed as a potential conflict of interest.

## Abbreviations

BGSC: *Bacillus* Genetic Stock Center
NRPS: non-ribosomal polypeptide synthetases
PKS: polyketide synthetases
RiPP: Ribosomally synthesized and post-translationally modified peptides
PGPB: plant growth promoting bacteria
Bcsl: *Bacillus cereus sensu lato*
Bt: *Bacillus thuringiensis*
Bw: *Bacillus weihenstephanensis*
Ba: *Bacillus anthracis*
Bm: *Bacillus mycoides*
Bp: *Bacillus pseudomycoides*
Bc: *Bacillus cereus*
Bcyt: *Bacillus cytotoxicus*.
