# Supplementary material for: Bacillus thuringiensis and Bacillus weihenstephanensis Inhibit the Growth of Phytopathogenic Verticillium Species
Source: Front Microbiol. 2017 Jan 18;7:2171. doi: 10.3389/fmicb.2016.02171 (PMC5241308; doi:10.3389/fmicb.2016.02171)
Supplement: Supplementary file 1 [file DataSheet1.DOCX]

Supplementary Material

***Bacillus thuringiensis* and *Bacillus weihenstephanensis* inhibit the growth of phytopathogenic *Verticillium* species**

**Hollensteiner J^1^, Wemheuer F^1^, Harting R^2^, Kolarzyk A^2,3^, Diaz-Valerio SM^1^, Poehlein A^1^, Brzuszkiewicz E^1^, Nesemann K^2^, Braus-Stromeyer SA^2^, Braus GH^2^, Daniel R^1^ and Liesegang H^1*^**

*** Correspondence:** Liesegang H: [hlieseg@gwdg.de](mailto:hlieseg@gwdg.de)

# Supplementary Tables

Supplementary Table 1. Primer pairs used in MLST experiments according to (Priest et al., 2004).

| Gene | Forward primer (5’-3’) | Reverse primer (5’-3’) |
| --- | --- | --- |
| *glpF* | GCGTTTGTGCTGGTGTAAGT | CTGCAATCGGAAGGAAGAAG |
| *gmk* | ATTTAAGTGAGGAAGGGTAGG | GCAATGTTCACCAACCACAA |
| *ilvD* | GCAGAGATTAAAGATAAGGA | GTTACCATTTGTGCATAACGC |
| *pta* | GCAGAGCGTTTAGCAAAAGAA | TGCAATGCGAGTTGCTTCTA |
| *purH* | CTGCTGCGAAAAATCACAAA | CTCACGATTCGCTGCAATAA |
| *pycA* | GCGTTAGGTGGAAACGAAAG | CGCGTCCAAGTTTATGGAAT |
| *tpi* | GCCCAGTAGCACTTAGCGAC | CCGAAACCGTCAAGAATGAT |

Supplementary Table 3. Exclusion of *Bacillus anthracis* virulence factors in isolates.

| Strain | Genome | Anthrax prophages | PlcR-nonsense mutation | Lethal factor | Protective antigen | DnaA_Ba | DnaA_Bt | DnaA_Bc |
| --- | --- | --- | --- | --- | --- | --- | --- | --- |
| Bt GOE1 | 5,359,363 | No hits | 285 AS, no differences to *B. cereus* group | absent | absent | -^*^ | -^*^ | -^*^ |
| Bt GOE2 | 5,373,416 | No hits | 285 AS, no differences to *B. cereus* group | absent | absent | -^*^ | -^*^ | -^*^ |
| Bt GOE3 | 5,347,504 | No hits | 285 AS, no differences to *B. cereus* group | absent | absent | -^*^ | -^*^ | -^*^ |
| Bt GOE4 | 6,008,382 | No hits | 285 AS, no differences to *B. cereus* group | absent | absent | -^*^ | -^*^ | -^*^ |
| Bt GOE5 | 5,772,687 | No hits | 285 AS, no differences to *B. cereus* group | absent | absent | -^*^ | -^*^ | -^*^ |
| Bt GOE6 | 5,857,591 | No hits | 285 AS, no differences to *B. cereus* group | absent | absent | -^*^ | -^*^ | -^*^ |
| Bt GOE7 | 5,976,466 | No hits | 285 AS, no differences to *B. cereus* group | absent | absent | -^*^ | -^*^ | -^*^ |
| Bw GOE1 | 5,613,589 | No hits | no PlcR, related rap/phr systems | absent | absent | 96,19 | 96,19 | 100 |
| Bw GOE2 | 5,581,838 | No hits | no PlcR, related rap/phr systems | absent | absent | 96,19 | 96,19 | 100 |
| Bw GOE3 | 5,644,666 | No hits | no PlcR, related rap/phr systems | absent | absent | 96,19 | 96,19 | 100 |
| Bw GOE4 | 5,597,134 | No hits | no PlcR, related rap/phr systems | absent | absent | 96,19 | 96,19 | 100 |
| Bw GOE5 | 5,589,822 | No hits | no PlcR, related rap/phr systems | absent | absent | 96,19 | 96,19 | 100 |
| Bw GOE6 | 5,632,050 | No hits | no PlcR, related rap/phr systems | absent | absent | 96,19 | 96,19 | 100 |
| Bw GOE7 | 5,772,687 | No hits | no PlcR, related rap/phr systems | absent | absent | 96,19 | 96,19 | 100 |
| Bw GOE8 | 5,851,749 | No hits | no PlcR, related rap/phr systems | absent | absent | 95,96 | 95,96 | 100 |
| Bw GOE9 | 5,846,892 | No hits | no PlcR, related rap/phr systems | absent | absent | 95,96 | 95,96 | 100 |
| Bw GOE10 | 5,823,784 | No hits | no PlcR, related rap/phr systems | absent | absent | 95,96 | 95,96 | 100 |
| Bw GOE11 | 5,625,374 | No hits | no PlcR, related rap/phr systems | absent | absent | 96,19 | 96,19 | 100 |
| Bw GOE12 | 5,674,772 | No hits | no PlcR, related rap/phr systems | absent | absent | 96,19 | 96,19 | 100 |
| Bw GOE13 | 5,642,420 | No hits | no PlcR, related rap/phr systems | absent | absent | 96,19 | 96,19 | 100 |

^-*^DnaA, not discriminative between Ba, Bt and Bc.

Supplementary Table 4. Growth effects on *Verticillium dahliae* JR2 and *Verticillium longisporum* 43 by bacterial strains.

| Bacterial Strain | Inhibition zone against  *Verticillium dahliae* JR2 plates | | Inhibition zone against *Verticillium longisporum* 43 plates | |
| --- | --- | --- | --- | --- |
|  | Media | | | |
|  | LB | PDM | LB | PDM |
| *E. coli* DH5α | - | - | - | - |
| 4D2* | b | ++a | +b | #a |
| 4Q1* | b | #a | b | a |
| Bt GOE1 | b | ++a | b | a |
| Bt GOE2 | b | ++a | b | a |
| Bw GOE1 | - | - | - | - |
| Bt GOE3 | b | ++a | b | #a |
| Bw GOE2 | - | - | - | - |
| Bt GOE4 | ++b | ++a | ++b | #a |
| Bt GOE5 | b | ++a | b | #a |
| Bt GOE6 | ++b | - | +++b | - |
| Bw GOE3 | - | - | - | - |
| Bw GOE4 | - | - | - | - |
| Bw GOE5 | - | - | - | - |
| Bw GOE6 | - | - | - | - |
| Bw GOE7 | - | - | - | - |
| Bw GOE8 | c | - | c | - |
| Bw GOE9 | c | - | c | - |
| Bw GOE10 | c | - | c | - |
| Bw GOE11 | - | - | - | - |
| Bw GOE12 | - | - | - | - |
| Bw GOE13 | - | - | - | - |
| Bt GOE7 | b | ++a | b | +a |
| MYBT18246** | b | - | b | - |
| Bt18247** | b | - | b | - |
| Bt18679** | b | ++a | b | #a |
| Btt*** | b | ++a | b | a |

Symbols refer to fungal and bacterial growth: inhibition zone (mm) observed among growing fungal cells: -, absence of effect; a) white air-mycelium/strong melanization; b) strong biofilm formation of bacteria; c) bacteria build hyphae-like structures. Strength of inhibitory effect: + = 0.1-2.4 mm; ++ = 2.5-5.3 mm; +++ = 5.4-9 mm, or ++++ = >9.1- mm; *insecticidal strains; **nematocidal strains; # = A phenotypic effect was observed, but no measurable inhibition was quantified due to study limitations. Strain details and abbreviations are depicted in Table 2.

# Supplementary Figures

# Supplementary Figure 1. Average rarefaction curves calculated for tomato root samples. Rarefaction curves showing diversity of OTUs at 3% genetic dissimilarity associated with tomato root soil.

Supplementary Figure 2. Inhibition zones of selected bacterial strains against *V. dahliae* JR2 plates. Inhibition is hereby defined by a clear zone around the bacteria where *V. dahliae JR2* does not exhibit growth (sometimes with a slight formation of microsclerotia). Zones have been measured by the distance of the bacteria inoculum and the boundary of the *V. dahliae* JR2 background.

Supplementary Figure 3. Inhibition zones of selected bacterial strains against *V. longisporum*43 plates. Inhibition is hereby defined by a clear zone around the bacteria where *V. longisporum* 43 does not exhibit growth. Zones have been measured by the distance of the bacteria inoculum and the boundary of the *V. longisporum* 43 background. Formation of microsclerotia, the melanized resting structures of the fungus, can be observed as dark rings around the bacteria.

References

Priest, F. G., Barker, M., Baillie, L. W. J., Holmes, E. C., and Maiden, M. C. J. (2004). Population Structure and Evolution of the Bacillus cereus Group †. *Society* 186, 7959–7970. doi:10.1128/JB.186.23.7959.
