# Supplementary figures and images for: Bacillus thuringiensis and Bacillus weihenstephanensis Inhibit the Growth of Phytopathogenic Verticillium Species
Source: Front Microbiol. 2017 Jan 18;7:2171. doi: 10.3389/fmicb.2016.02171 (PMC5241308; doi:10.3389/fmicb.2016.02171)

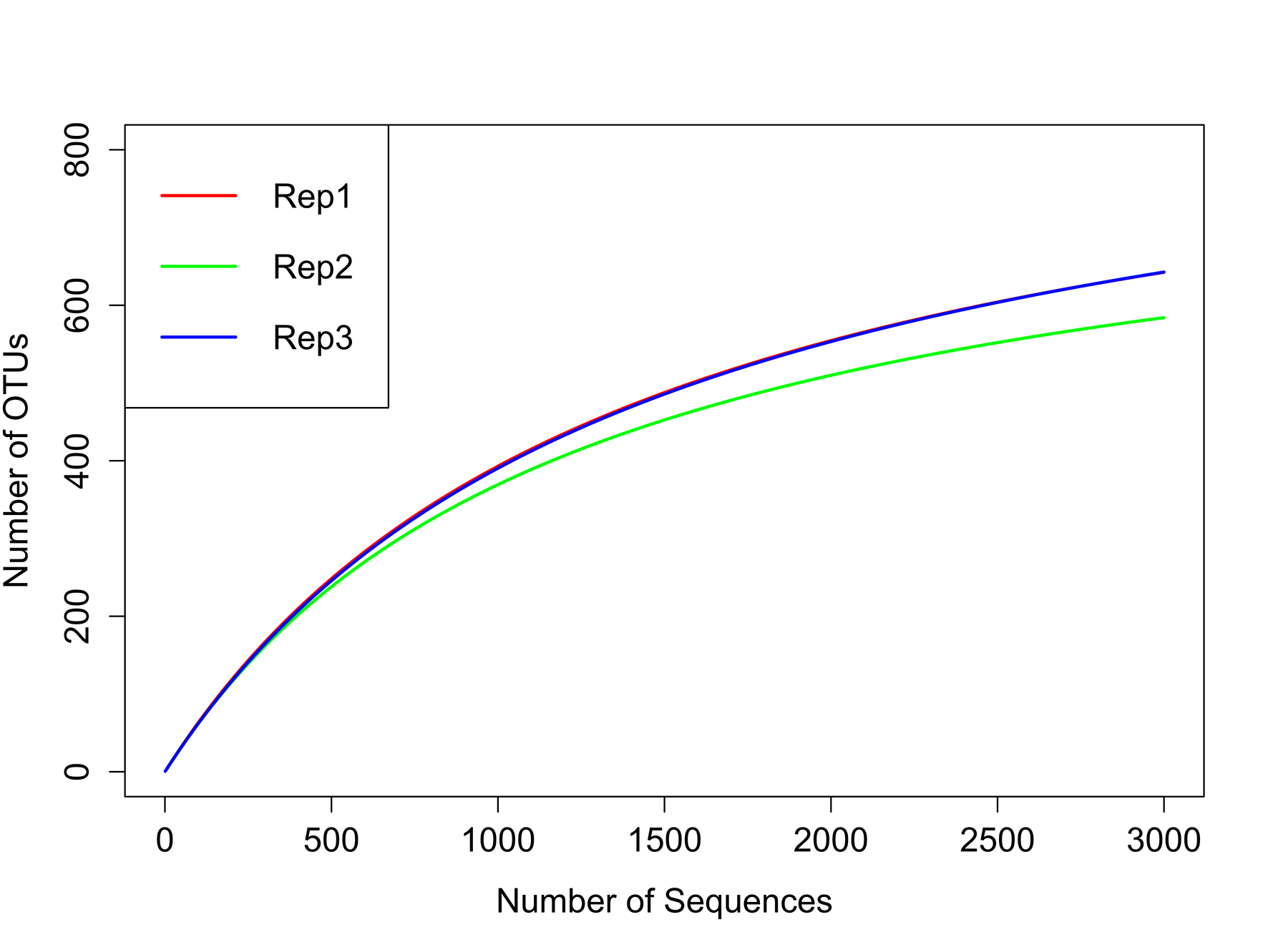

Supplement: Supplementary file 4 [file Image1.TIF]

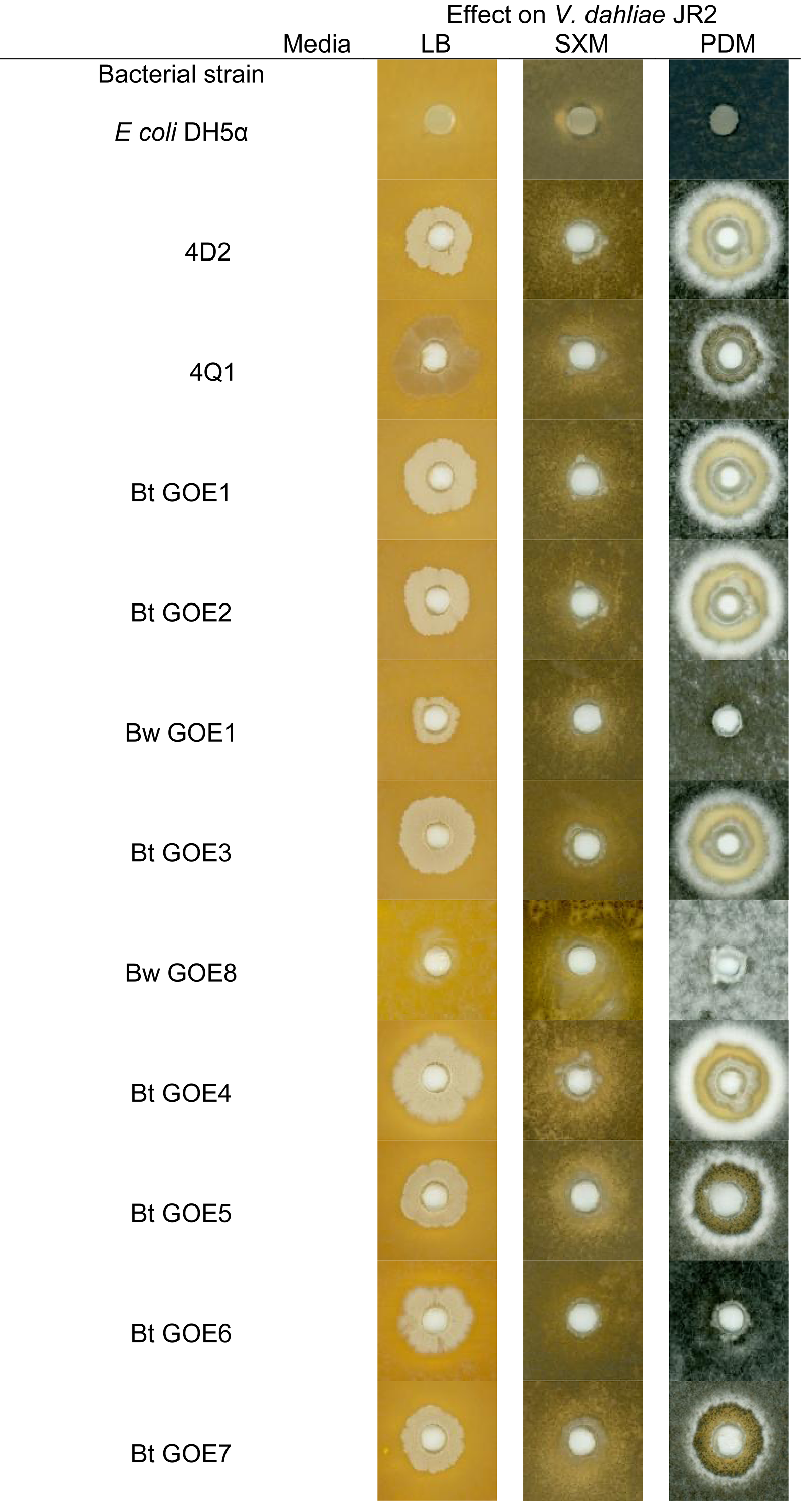

Supplement: Supplementary file 5 [file Image2.TIF]

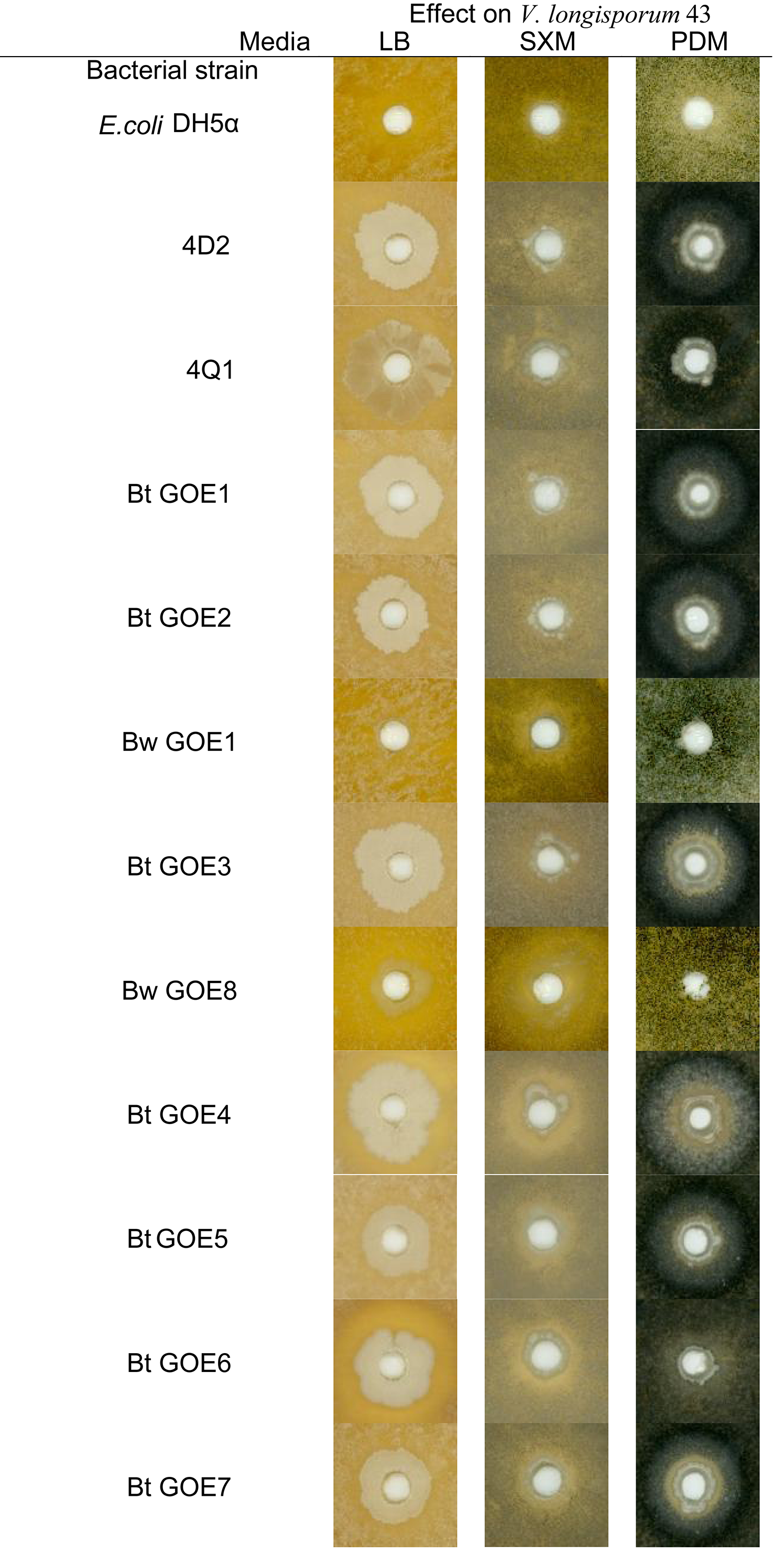

Supplement: Supplementary file 6 [file Image3.TIF]
